# Flexible Polymer Hydrogel Materials for Next-Generation Wearable Energy Storage Technologies

**DOI:** 10.3390/gels11120999

**Published:** 2025-12-11

**Authors:** Thirukumaran Periyasamy, Shakila Parveen Asrafali, Jaewoong Lee

**Affiliations:** Department of Fiber System Engineering, Yeungnam University, Gyeongsan 38541, Republic of Korea

**Keywords:** polymer hydrogels, porous nanostructures, fabrication method, flexible and wearable, energy storage

## Abstract

The rapid advancement of wearable technology has created an increasing demand for efficient, high-performance energy storage systems that also offer key characteristics such as flexibility, lightweight, and durability. Among the emerging materials, polymer hydrogels have garnered significant attention due to their unique combination of viscoelasticity, low density, and tunable porous nanostructures. These materials exhibit adaptable surface and structural properties, making them promising candidates for next-generation flexible and wearable energy storage devices. This work provides an overview of recent progress and innovations in the application of polymer hydrogels for the development of flexible energy storage systems. The intrinsic three-dimensional architecture and porous morphology of these hydrogels offer a versatile platform for constructing high-performance supercapacitors, rechargeable batteries, and personal thermal management devices. Various types of polymer hydrogels and their principal fabrication methods are discussed in detail, along with the structural factors that influence their electrochemical and mechanical performance. Furthermore, recent advancements in integrating polymer hydrogel materials into wearable and flexible technologies—such as energy storage devices, thermal regulation systems, and multifunctional energy platforms—are comprehensively reviewed and analyzed.

## 1. Introduction

The exponential growth of portable and wearable electronic technologies over the last two decades has created an unprecedented demand for innovative energy storage solutions that can accommodate the unique requirements of these devices. Unlike conventional rigid electronics, wearable technologies must conform to the dynamic nature of the human body, enduring repeated mechanical deformations including bending, stretching, twisting, and compression while maintaining consistent electrochemical performance. This paradigm shift in electronic device architecture necessitates a fundamental reimagining of energy storage systems, moving away from traditional rigid batteries and capacitors toward flexible, stretchable, and mechanically robust alternatives that can seamlessly integrate with the human form [1,2,3,4,5].

**Figure 1:**
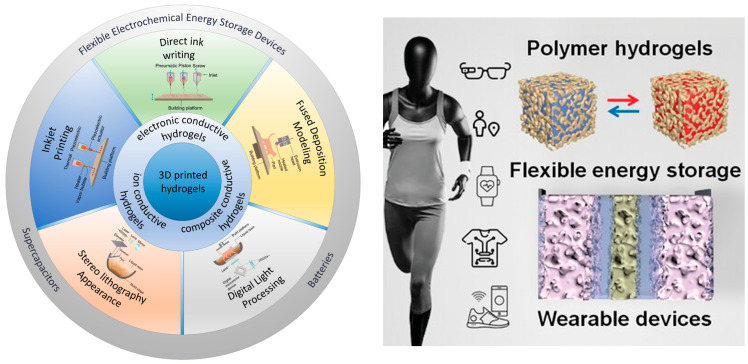
Key Physicochemical Characteristics of Polymer Hydrogels and Their Roles in Energy-Storage Applications [13].

Polymer hydrogels have emerged as exceptionally promising materials for addressing the multifaceted challenges associated with wearable energy storage systems. These three-dimensional networked structures, composed of hydrophilic polymer chains that retain substantial quantities of water or electrolyte solutions within their porous architecture, offer a unique combination of properties that are particularly advantageous for flexible energy storage applications (Figure 1). The inherent viscoelastic nature of hydrogels enables them to undergo significant deformations without catastrophic failure, while their high porosity and interconnected network structure facilitate efficient ion transport essential for electrochemical energy storage processes. Furthermore, the tunable chemical and physical properties of polymer hydrogels allow for precise optimization of characteristics such as mechanical strength, ionic conductivity, electrochemical stability, and interfacial compatibility with electrode materials [6,7,8].

The versatility of polymer hydrogels extends across multiple components of energy storage devices. They can function as flexible electrodes with high surface areas for charge storage, as solid or quasi-solid electrolytes that eliminate leakage concerns associated with liquid electrolytes, as separators that prevent short circuits while allowing ion transport, and as binders that maintain electrode integrity during mechanical deformation. In thermal energy storage applications, polymer hydrogels serve as shape-stabilizing matrices for phase change materials, providing structural support while facilitating heat transfer. This multifunctionality, combined with the potential for self-healing properties and biocompatibility in certain formulations, positions polymer hydrogels as cornerstone materials for next-generation wearable energy storage technologies [9,10,11,12].

Current research in polymer hydrogel-based energy storage systems has demonstrated remarkable achievements, including supercapacitors with energy densities approaching those of batteries, batteries with exceptional flexibility and cycle life, and thermal management systems with high heat storage capacity and rapid charging/discharging capabilities. However, significant challenges remain in areas such as achieving simultaneously high energy density and mechanical robustness, ensuring long-term electrochemical and mechanical stability, developing environmentally sustainable and biocompatible formulations, and scaling up production processes for commercial viability. This review provides a comprehensive examination of recent advances in polymer hydrogel materials for wearable energy storage applications, analyzing the relationships between hydrogel structure, properties, and performance, and identifying critical research directions for future development.

## 2. Hydrogels

Hydrogels represent a distinctive class of soft materials characterized by their three-dimensional polymer network structures capable of absorbing and retaining large volumes of water or aqueous solutions, typically exceeding 40 percent of their total weight, while maintaining structural integrity. The fundamental architecture of hydrogels consists of hydrophilic polymer chains interconnected through physical or chemical crosslinks, creating a porous network with high water content that imparts unique properties including softness, flexibility, and biocompatibility. The classification of hydrogels can be approached from multiple perspectives, including the chemical nature of the constituent materials, the type of crosslinking mechanisms, the source of polymer components, and the intended application domain [14,15,16,17].

From a compositional standpoint, hydrogels can be broadly categorized into organic hydrogels, inorganic hydrogels, organic-inorganic hybrid hydrogels, and composite hydrogels. Organic hydrogels, which constitute the primary focus of this review, are formed from carbon-based polymeric materials and can be further subdivided into polymer hydrogels and carbon hydrogels. Polymer hydrogels are constructed from traditional polymeric macromolecules, either natural or synthetic, that are crosslinked to form three-dimensional networks. These materials exhibit a wide range of properties depending on the chemical structure of the polymer backbone, the nature and density of crosslinks, and the presence of functional groups that influence hydrophilicity and interaction with solvents and ions.

Carbon hydrogels, in contrast, are assembled from carbon-based nanomaterials such as graphene, graphene oxide, carbon nanotubes, or carbonized polymer precursors. These materials typically exhibit superior electrical and thermal conductivity compared to conventional polymer hydrogels, making them particularly attractive for energy storage applications where efficient electron and heat transport are critical. However, carbon hydrogels often lack the mechanical flexibility and biocompatibility of polymer hydrogels, and their synthesis typically requires more complex processing conditions including high-temperature treatments or chemical reduction steps. Inorganic hydrogels are constructed from inorganic materials such as silica, metal oxides, or layered double hydroxides. While these materials can exhibit interesting properties including high thermal stability and unique catalytic activities, their application in wearable energy storage is limited by brittleness and processing challenges. Hybrid and composite hydrogels, which combine organic and inorganic components or incorporate various types of fillers and additives into polymer matrices, represent a strategy for achieving synergistic property combinations that cannot be obtained from single-component systems. These multicomponent hydrogels can be engineered to exhibit enhanced electrical conductivity, mechanical strength, thermal stability, and electrochemical performance while retaining the flexibility and processability advantages of polymer-based systems [18,19,20,21].

The distinction between these hydrogel categories has important implications for energy storage applications. Organic polymer hydrogels generally offer superior mechanical flexibility and processability but may require enhancement of electrical conductivity through incorporation of conductive additives or use of intrinsically conductive polymers. Carbon hydrogels provide excellent electrical conductivity but may sacrifice mechanical robustness and require careful optimization of synthesis conditions to achieve desired structural properties. Hybrid and composite approaches offer pathways to balanced property profiles but introduce additional complexity in materials design and processing. Understanding these fundamental differences is essential for rational selection and design of hydrogel materials for specific energy storage applications.

## 3. Polymer Hydrogels

Polymer hydrogels occupy a central position in the landscape of materials for wearable energy storage applications due to their unique combination of properties that can be tailored through judicious selection of polymer chemistry, crosslinking mechanisms, and processing conditions. These materials can be classified based on multiple criteria, including the origin of the polymer component, the nature of the crosslinking interactions, the resulting mechanical and electrochemical properties, and the intended application. Understanding the relationships between these classification schemes and the resulting material properties is essential for designing polymer hydrogels optimized for specific energy storage requirements. The distinction between natural and synthetic polymer hydrogels represents a fundamental classification with significant implications for material properties and applications. Natural polymer hydrogels are derived from biopolymers such as polysaccharides, proteins, and nucleic acids obtained from renewable biological sources. Common examples include chitosan, alginate, cellulose, gelatin, collagen, and hyaluronic acid. These materials typically exhibit excellent biocompatibility, biodegradability, and low toxicity, making them attractive for biomedical applications and environmentally conscious technologies. However, natural polymer hydrogels often suffer from limitations including batch-to-batch variability, limited mechanical strength, susceptibility to enzymatic degradation, and low intrinsic electrical conductivity, which can restrict their direct application in energy storage devices [3,9,18].

Synthetic polymer hydrogels are prepared from man-made polymers synthesized through controlled polymerization reactions. Common synthetic polymers used for hydrogel fabrication include polyacrylamide, polyvinyl alcohol, polyethylene glycol, poly(N-isopropylacrylamide), and various polyelectrolytes. Synthetic polymers offer advantages including well-defined chemical structures, reproducible properties, tunable molecular weights and architectures, and the ability to incorporate functional groups that impart specific properties such as electrical conductivity or stimuli-responsiveness. The controlled synthesis of synthetic polymers enables precise engineering of hydrogel properties, although concerns regarding biocompatibility, biodegradability, and environmental impact must be carefully considered. A particularly important subclass of synthetic polymer hydrogels for energy storage applications consists of intrinsically conductive polymers including polyaniline, polypyrrole, polythiophene, and poly(3,4-ethylenedioxythiophene). These conjugated polymers possess delocalized π-electron systems that enable electrical conductivity and electrochemical activity, making them ideal candidates for electrodes in supercapacitors and batteries. Conductive polymer hydrogels can be synthesized through oxidative polymerization of appropriate monomers under conditions that promote simultaneous polymerization and gelation, resulting in three-dimensional networked structures with both mechanical flexibility and electrical conductivity. The electrochemical properties of conductive polymer hydrogels can be further tuned through control of polymerization conditions, doping levels, and incorporation of additional functional components [22,23,24,25].

### 3.1. Fabrication of Polymer Hydrogels

The synthesis and fabrication methods employed for polymer hydrogel preparation exert profound influences on the resulting material properties, including network structure, mechanical characteristics, porosity, and electrochemical performance. The selection of an appropriate fabrication strategy depends on multiple factors including the chemical nature of the polymer components, the desired crosslinking density and architecture, the intended application requirements, and practical considerations such as scalability and cost. Fabrication methods can be broadly classified based on the type of crosslinking mechanism employed: chemical crosslinking, physical crosslinking, and double crosslinking approaches that combine both mechanisms [26].

#### 3.1.1. Chemical Cross-Linking

Chemical crosslinking involves the formation of covalent bonds between polymer chains, creating a permanent three-dimensional network structure. This approach can be implemented through multiple pathways, including direct polymerization of multifunctional monomers, crosslinking of preformed polymer chains using bifunctional or multifunctional crosslinking agents, or radiation-induced crosslinking through exposure to ultraviolet light, gamma radiation, or electron beams. The resulting chemically crosslinked hydrogels typically exhibit robust mechanical properties, good dimensional stability, and resistance to dissolution in solvents, although the permanent nature of covalent crosslinks generally precludes self-healing capabilities.

**Figure 2:**
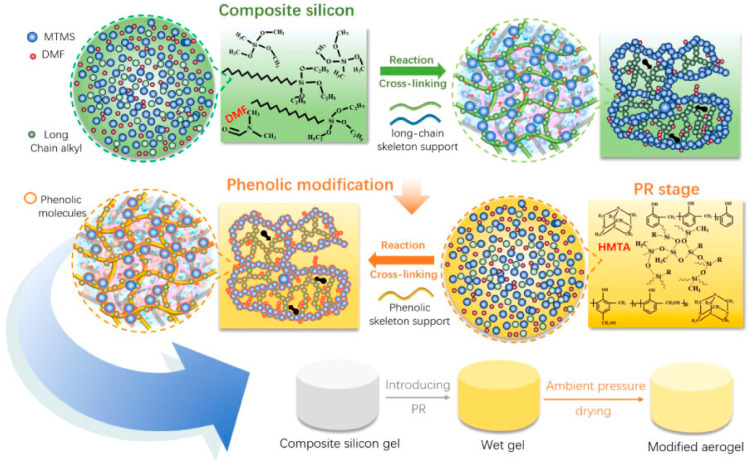
Schematic Illustration of the Reaction Mechanism of the MTMS/Hexadecyltrimethoxysilane Aerogel [32].

Radical polymerization represents one of the most versatile methods for synthesizing chemically crosslinked hydrogels. In this approach, vinyl monomers containing polymerizable double bonds are converted into crosslinked networks through free radical chain-growth polymerization in the presence of multifunctional crosslinking agents (Figure 2). The polymerization can be initiated thermally using radical initiators such as ammonium persulfate or azobisisobutyronitrile, or photochemically using photoinitiators activated by ultraviolet or visible light. The degree of crosslinking, which critically influences mechanical properties and swelling behavior, can be controlled by adjusting the ratio of crosslinker to monomer, the total monomer concentration, and the polymerization conditions [27,28,29,30,31,32].

Condensation polymerization provides an alternative route to chemically crosslinked hydrogels through step-growth mechanisms involving the reaction of complementary functional groups. For example, polyvinyl alcohol can be crosslinked using dialdehydes such as glutaraldehyde, which react with hydroxyl groups on adjacent polymer chains to form acetal linkages. Similarly, polyacrylic acid can be crosslinked through esterification reactions with polyols or through amidation with diamines. Condensation crosslinking typically proceeds under milder conditions than radical polymerization and allows for greater control over network architecture, although reaction times may be longer and byproducts such as water or small molecules may be generated. Click chemistry reactions, characterized by high efficiency, specificity, and mild reaction conditions, have emerged as powerful tools for fabricating chemically crosslinked hydrogels with well-defined structures. The copper-catalyzed azide-alkyne cycloaddition reaction represents the prototypical click reaction and has been widely employed for hydrogel synthesis. By functionalizing polymer chains with azide and alkyne groups, rapid gelation can be achieved under physiological conditions in the presence of copper catalysts. Other click reactions including thiol-ene additions, Diels-Alder reactions, and oxime formations have also been successfully applied to hydrogel fabrication, offering diverse chemical toolboxes for creating networks with specific properties [33,34,35].

Enzyme-catalyzed crosslinking represents a biologically inspired approach that utilizes enzymatic reactions to form covalent bonds between polymer chains. Horseradish peroxidase, for example, can catalyze the oxidative coupling of phenolic groups on polymer chains in the presence of hydrogen peroxide, leading to rapid gelation under mild conditions compatible with biological systems. Transglutaminase can catalyze the formation of isopeptide bonds between glutamine and lysine residues on protein-based polymers. Enzyme-catalyzed crosslinking offers advantages including high specificity, operation under physiological conditions, and compatibility with biological molecules, although enzyme cost and stability may present practical limitations.

#### 3.1.2. Physical Cross-Linking

**Figure 3:**
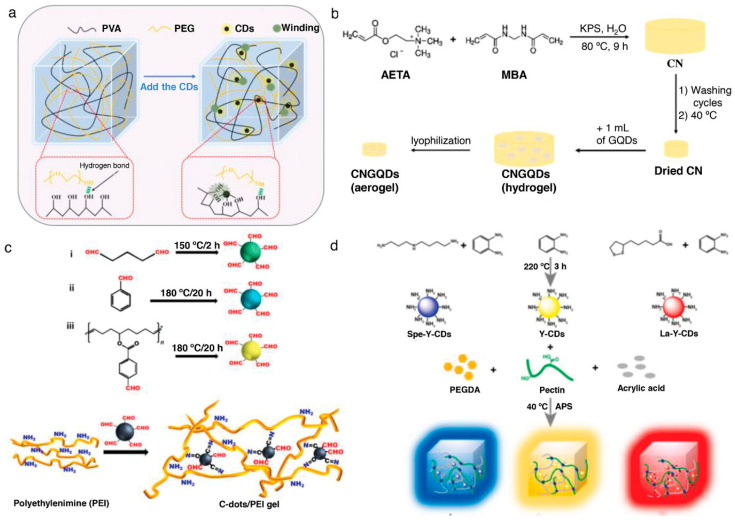
(**a**) Mechanism diagram of the PVA–PEG/CDs hydrogel. (**b**) Preparation process of the GQD-based hydrogel. (**c**) Synthesis of aldehyde-CDs from various aldehyde precursors and formation of C-dot/PEI gels via Schiff base reaction. (**d**) Design strategy of multi-color-emitting, tough, and antibacterial hydrogels using cationic CDs, pectin, Ac, PEGDA, and APS [36].

Physical crosslinking relies on non-covalent interactions between polymer chains to create three-dimensional network structures. Unlike chemical crosslinking, physical crosslinks are typically reversible and can be disrupted and reformed in response to environmental changes such as temperature, pH, or ionic strength. This reversibility enables unique properties including self-healing, injectability, and stimuli-responsiveness, which can be advantageous for certain energy storage applications. However, physically crosslinked hydrogels may exhibit lower mechanical strength and greater susceptibility to dissolution or disintegration compared to chemically crosslinked counterparts (Figure 3a–d) [19,28,36].

Hydrogen bonding represents one of the most common physical crosslinking mechanisms in polymer hydrogels. Polymers containing hydrogen bond donors such as hydroxyl, amine, or carboxylic acid groups and acceptors such as carbonyl or ether oxygen atoms can form extensive hydrogen bonding networks that stabilize three-dimensional structures. Polyvinyl alcohol hydrogels prepared through freeze–thaw cycling represent a classic example, where repeated freezing and thawing promotes the formation of crystalline regions stabilized by hydrogen bonding between hydroxyl groups on adjacent chains. The strength and reversibility of hydrogen bonding networks depend on factors including the density and spatial arrangement of functional groups, the presence of competing interactions with solvent molecules, and temperature. Hydrophobic interactions provide another important mechanism for physical crosslinking, particularly in amphiphilic polymers containing both hydrophilic and hydrophobic segments. In aqueous environments, hydrophobic segments tend to aggregate to minimize contact with water, forming physical crosslinks that stabilize the network structure. This phenomenon is exploited in the design of thermoreversible hydrogels based on block copolymers such as Pluronic or poly(N-isopropylacrylamide), which exhibit temperature-dependent gelation due to the balance between hydrophobic interactions and polymer-solvent interactions. Hydrophobic crosslinking can provide good mechanical properties while maintaining reversibility, although the stability of these networks may be sensitive to temperature and the presence of organic solvents or surfactants [37,38,39,40,41].

Ionic interactions between charged polymer chains and multivalent counterions can lead to physical crosslinking through the formation of ionic bridges or electrostatic complexes. Alginate hydrogels crosslinked by calcium ions represent a widely studied example, where carboxylate groups on alginate chains coordinate with calcium ions to form egg-box structures that stabilize the gel network. Similarly, chitosan can be crosslinked by polyanions such as tripolyphosphate through electrostatic interactions between protonated amine groups and phosphate groups. Ionic crosslinking offers advantages including rapid gelation under mild conditions and reversibility through changes in pH or ionic strength, although the mechanical properties and stability of ionically crosslinked hydrogels may be limited.

Crystallization-induced gelation represents a physical crosslinking mechanism where crystalline regions formed by ordered packing of polymer chains serve as physical crosslinks. This phenomenon is commonly observed in semicrystalline polymers such as polyvinyl alcohol, where crystalline domains act as junction points connecting amorphous regions. The extent of crystallization, and thus the crosslink density, can be controlled through processing conditions such as the number of freeze–thaw cycles, annealing temperature and time, or polymer concentration. Crystalline crosslinks generally provide good mechanical strength and stability while maintaining some degree of reversibility at elevated temperatures where crystals can melt [42,43,44,45].

#### 3.1.3. Double Cross-Linking

**Figure 4:**
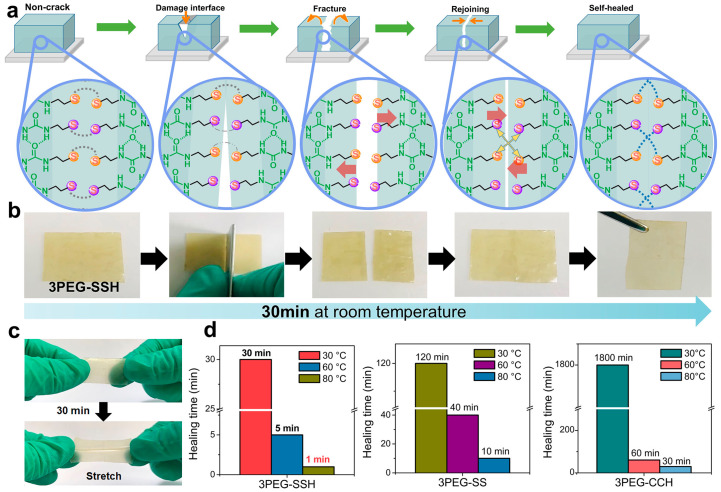
Self-healing characteristics of SHSPEs, including (**a**) healing mechanism, (**b**) room-temperature healing, (**c**) mechanical recovery, and (**d**) healing kinetics across samples [46].

Double crosslinking strategies combine both chemical and physical crosslinking mechanisms within a single hydrogel network, creating hierarchical structures that can exhibit synergistic property combinations not achievable through either mechanism alone. The integration of permanent covalent crosslinks with reversible physical crosslinks enables the design of materials that simultaneously possess mechanical robustness, toughness, and self-healing capabilities. This approach has gained significant attention for energy storage applications where materials must withstand repeated mechanical deformations while maintaining structural integrity and electrochemical performance. The classic double network hydrogel architecture consists of two interpenetrating polymer networks with contrasting properties: a rigid, densely crosslinked first network that provides mechanical strength, and a soft, loosely crosslinked second network that contributes to toughness and energy dissipation. During mechanical deformation, the rigid network undergoes controlled fracture, dissipating energy and preventing catastrophic failure, while the soft network maintains overall structural integrity (Figure 4a–d). This sacrificial bond mechanism enables double network hydrogels to achieve exceptional toughness and fracture resistance while retaining flexibility. The synthesis typically involves sequential polymerization steps, where the first network is formed and swollen with monomers for the second network before initiating the second polymerization [42,43,44,45,46,47,48,49,50].

**Figure 5:**
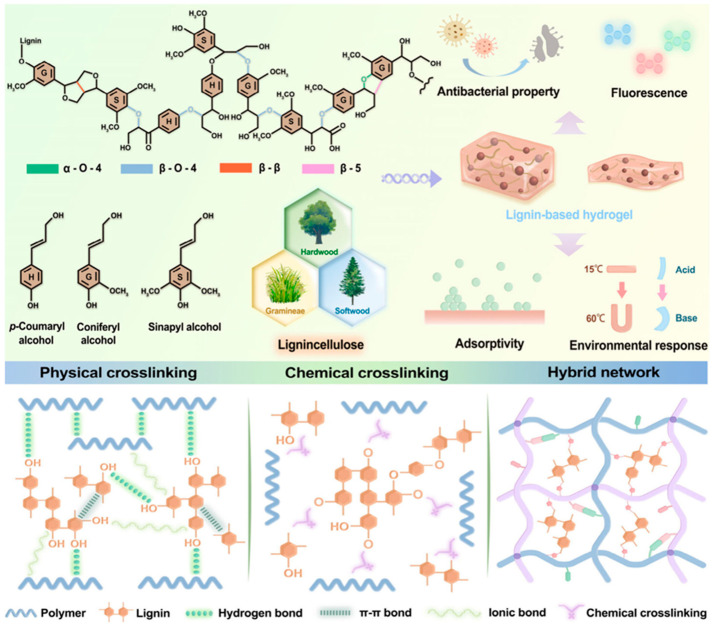
Lignin Macromolecular Architecture, Functional Characteristics of Lignin-Based Hydrogels, and Key Cross-Linking Mechanisms [50].

Polyampholyte hydrogels represent another class of double crosslinked materials where the same polymer chains contain both cationic and anionic groups that can form reversible ionic crosslinks in addition to permanent covalent crosslinks. The random distribution of oppositely charged groups along the polymer backbone leads to the formation of ionic bonds between chains, creating a physically crosslinked network superimposed on the covalent network (Figure 5). These materials can exhibit remarkable mechanical properties including high strength, toughness, and self-recovery, along with interesting stimuli-responsive behavior related to the pH-dependent ionization of charged groups. The balance between covalent and ionic crosslinks can be tuned through control of polymer composition and synthesis conditions [51,52,53,54,55,56,57,58,59,60]. Slide-ring hydrogels incorporate a unique double crosslinking concept where polymer chains are threaded through cyclic molecules such as cyclodextrins, which are then crosslinked to form a network with movable crosslinks. The ability of the cyclic molecules to slide along the polymer chains allows for stress redistribution and energy dissipation during deformation, resulting in excellent mechanical properties including high stretchability and toughness. Combined with covalent crosslinks, slide-ring hydrogels can achieve remarkable combinations of mechanical performance and self-healing capabilities. The synthesis involves complexation of polymers with cyclic molecules followed by crosslinking of the cyclic components, requiring careful control of stoichiometry and reaction conditions [61,62,63,64,65,66,67,68,69]. Table 1 summarizes the properties of polymer hydrogels obtained from different fabrication methods.

### 3.2. Structural and Morphological Properties of Polymer Hydrogels

The performance of polymer hydrogels in energy storage applications is intimately connected to their structural and morphological characteristics at multiple length scales, from molecular architecture to macroscopic morphology. Understanding and controlling these features is essential for optimizing material properties including ionic conductivity, electronic conductivity, mechanical flexibility, electrochemical stability, and interfacial compatibility with other device components. The key structural and morphological parameters include pore architecture and connectivity, crystallinity and molecular order, surface chemistry and wettability, and mechanical properties including elasticity and toughness [51,52,53].

#### 3.2.1. Morphology

The morphological characteristics of polymer hydrogels encompass the size, shape, distribution, and connectivity of pores within the three-dimensional network structure, as well as the architecture of the solid polymer framework. These features directly influence critical properties for energy storage including the accessible surface area for electrochemical reactions, the pathways available for ion transport, the mechanical response to deformation, and the ability to accommodate volume changes during charge–discharge cycles. Morphology is determined by multiple factors including the chemistry of the polymer and crosslinker, the synthesis method and conditions, the presence of additives or templates, and post-synthesis processing steps. Cellular or foam-like morphologies, characterized by closed or open-cell structures with spherical or polyhedral pores (pore size typically range between 50 nm and 100 µm) separated by thin polymer walls, are commonly observed in hydrogels synthesized through phase separation processes or using porogens that are subsequently removed. These structures can provide high porosity (70 to 95%) and large surface areas (up to 150 m^2^ g^−1^), although the connectivity between pores critically influences transport properties. Hydrogels with open-cell structures where pores are interconnected through windows or channels generally exhibit superior ion transport compared to closed-cell structures where pores are isolated. The pore size distribution, which can range from nanometers to micrometers depending on synthesis conditions, affects both the accessible surface area and the resistance to ion transport.

Fibrous morphologies, where the polymer network consists of interconnected fibers or filaments rather than continuous walls, represent another important structural motif. These architectures can arise from specific polymer chemistries that promote anisotropic aggregation, from directional processing methods such as electrospinning or aligned freezing, or from the use of fibrous templates. Fibrous hydrogels can exhibit advantageous properties including high porosity, anisotropic mechanical properties that can be exploited for specific applications, and efficient pathways for ion transport along fiber directions. The fiber diameter (50 nm–10 µm), orientation distribution, and degree of interconnection are key parameters that influence material performance. Colloidal or particulate morphologies, consisting of aggregated polymer nanoparticles or microparticles, can be formed through emulsion polymerization, precipitation processes, or sol–gel transitions. These structures exhibit unique properties related to the particle size and the nature of inter-particle connections, which can range from weak physical contacts to strong chemical bonds. Colloidal hydrogels may offer advantages including tunable porosity through control of particle size and packing, the ability to incorporate functional components within individual particles, and potential for self-healing through particle rearrangement. However, the mechanical properties and stability of these materials depend critically on the strength of inter-particle connections [54,55,56,57].

Hierarchical morphologies incorporating structural features at multiple length scales represent an advanced approach to achieving optimized property combinations. For example, hydrogels with macropores for efficient mass transport and mesopores or micropores for high surface area can provide both rapid ion transport and large electrochemical capacity. Similarly, aligned microchannels for directional ion transport combined with nanoscale porosity within channel walls can optimize both transport and storage properties. The fabrication of hierarchical structures typically requires sophisticated processing approaches including multi-step synthesis, templating with hierarchical templates, or processing methods that create structures at multiple scales.

#### 3.2.2. Microstructure

The microstructural characteristics of polymer hydrogels, encompassing molecular-level features such as crystallinity, chain orientation, crosslink density and distribution, and chemical composition heterogeneity, exert profound influences on macroscopic properties relevant to energy storage applications. These features determine fundamental material properties including elastic modulus, ionic and electronic conductivity, swelling behavior, and electrochemical stability. Understanding and controlling microstructure is essential for achieving desired performance characteristics and for establishing structure-property relationships that guide materials design.

**Figure 6:**
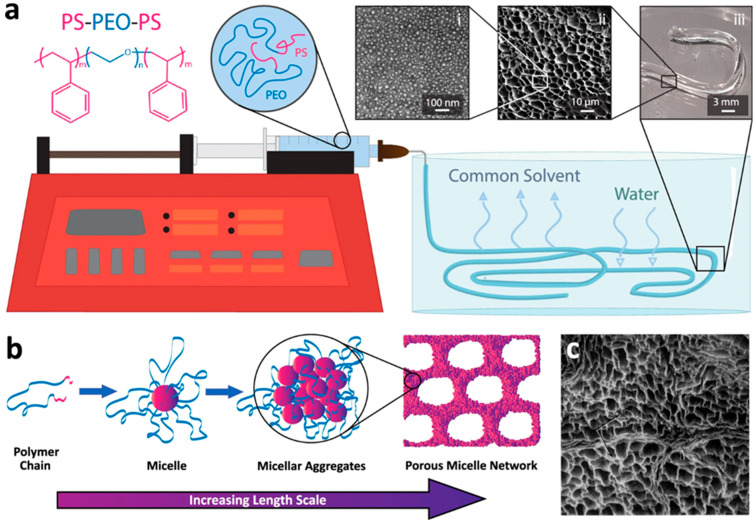
Processing and visualization of hierarchically structured hydrogels (**a**) Hydrogels exhibiting hierarchical order prepared using a rapid injection processing method; (**b**) Scheme representing the different levels of ordering during self-assembly; (**c**) Decellularized muscle tissue with natural hierarchical structure [61].

Crystallinity, defined as the fraction of polymer chains organized into ordered crystalline domains, significantly affects mechanical and transport properties of hydrogels. Semicrystalline hydrogels contain both crystalline regions that act as physical crosslinks and provide mechanical strength, and amorphous regions that contribute to flexibility and facilitate ion transport. The degree of crystallinity can be controlled through polymer chemistry, processing conditions such as cooling rate or annealing, and the presence of additives that promote or inhibit crystallization. Higher crystallinity generally increases mechanical strength and dimensional stability but may reduce ionic conductivity and flexibility, necessitating careful optimization for specific applications (Figure 6).

Chain orientation, which can be induced through processing methods such as stretching, shearing, or directional freezing, creates anisotropic microstructures with direction-dependent properties. Aligned hydrogels exhibit enhanced mechanical strength and toughness in the alignment direction, along with potentially improved ion transport along aligned pathways. For energy storage applications, controlled chain orientation can be exploited to create preferential ion transport pathways perpendicular to electrode surfaces, enhancing charge–discharge rates. However, anisotropic properties may also create challenges in applications requiring uniform performance in all directions [58,59,60,61].

Crosslink density and distribution critically influence the mechanical properties, swelling behavior, and transport characteristics of hydrogels. Higher crosslink densities generally increase mechanical strength and elastic modulus but reduce flexibility and swelling capacity. The spatial distribution of crosslinks, which can be homogeneous or heterogeneous depending on synthesis conditions, affects local mechanical properties and can create regions with different transport characteristics. Advanced synthesis approaches enabling control over crosslink density gradients or patterns can be used to create hydrogels with spatially varying properties tailored for specific functions. Chemical composition heterogeneity, arising from incomplete mixing of components, phase separation, or preferential distribution of certain species, can significantly impact hydrogel properties. In composite hydrogels containing conductive additives, the distribution of these additives determines the formation of percolating networks necessary for electronic conductivity. Similarly, the distribution of ionic species affects local ionic conductivity and electrochemical activity. Characterization and control of composition heterogeneity is essential for achieving reproducible properties and optimizing performance [62,63,64,65].

#### 3.2.3. Flexibility

**Figure 7:**
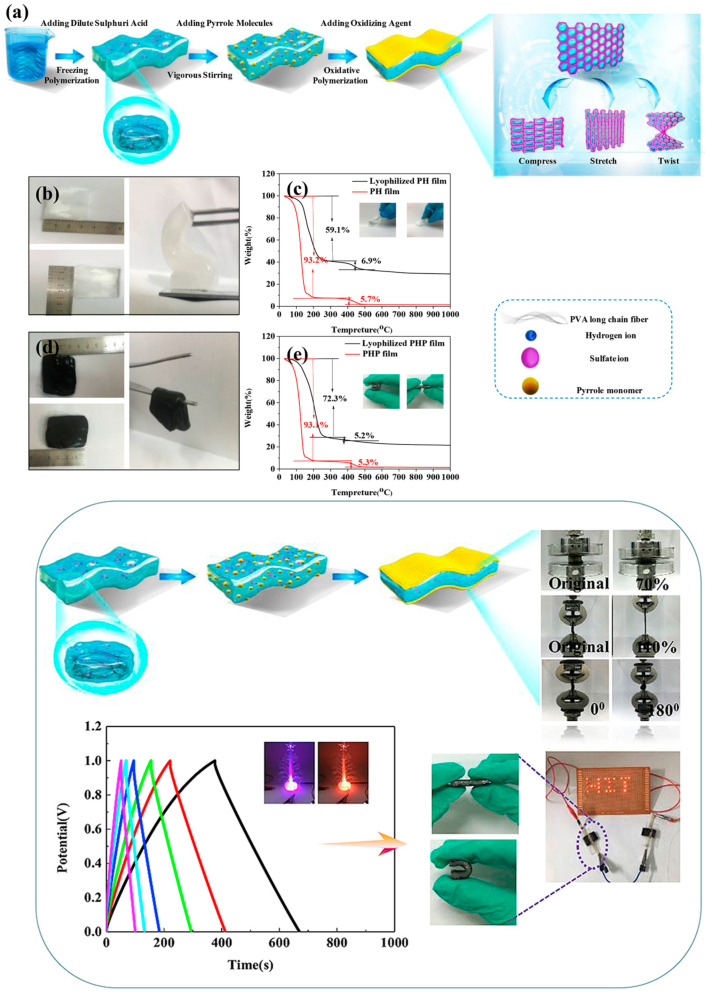
(**a**) Preparation process of the polypyrrole-polyvinyl alcohol/dilute sulphuric acid-polypyrrole (PHP) sandwiched device; (**b**) Digital photos of polyvinyl alcohol/dilute sulphuric acid (PH) hydrogel representing their soft behavior; (**c**) Thermogravimetric analysis (TGA) of swollen PH and lyophilized PH; (**d**) Digital photos of PHP hydrogel; (**e**) TGA curves of swollen PHP and lyophilized PHP. Structural Design, Physical Properties, and Thermal Analysis of the PHP Sandwiched Hydrogel Device [69].

Mechanical flexibility, encompassing the ability to undergo large deformations including bending, stretching, and twisting without failure or significant performance degradation, represents a critical requirement for wearable energy storage applications. The flexibility of polymer hydrogels is determined by multiple factors including polymer chemistry, crosslink type and density, water content, temperature, and the presence of additives. Achieving simultaneously high flexibility and mechanical robustness, along with stable electrochemical performance during deformation, represents a key challenge in hydrogel design for energy storage. The elastic modulus, which quantifies the stiffness or resistance to deformation, is a fundamental mechanical property that must be carefully tailored for wearable applications. Hydrogels for wearable devices typically require elastic moduli in the range of 10 kPa–10 MPa, significantly lower than rigid materials but sufficient to maintain structural integrity. The modulus can be controlled through crosslink density, with higher crosslinking generally increasing stiffness, and through water content, with higher swelling reducing modulus. The temperature dependence of modulus, related to the glass transition and melting behavior of polymer components, must also be considered for applications operating over a range of temperatures (Figure 7) [66,67,68,69].

Stretchability, defined as the maximum strain that can be sustained before failure, is particularly important for applications involving large deformations such as body-conforming devices. Highly stretchable hydrogels capable of elongations exceeding several hundred percent can be achieved through strategies including use of flexible polymer chemistries, incorporation of sliding crosslinks, creation of double network structures, and optimization of water content. However, high stretchability must be balanced against other requirements such as mechanical strength and dimensional stability. The ability to maintain electrochemical performance during stretching, requiring stable electrode-electrolyte interfaces and continuous ion transport pathways, represents an additional design challenge. Toughness, measured as the energy required to fracture a material, is critical for durability in applications involving repeated mechanical loading. Tough hydrogels can be designed through mechanisms including sacrificial bonds that dissipate energy during deformation, double network structures that combine rigid and soft components, and incorporation of energy-dissipating physical interactions. The toughness of hydrogels for energy storage applications must be sufficient to withstand not only mechanical deformations but also stresses arising from electrochemical processes such as volume changes during charging and discharging [70,71,72,73].

Fatigue resistance, or the ability to withstand repeated loading cycles without progressive damage accumulation, is essential for long-term durability of wearable devices. Hydrogels can experience fatigue through mechanisms including crack propagation, crosslink rupture, and gradual structural degradation. Improving fatigue resistance requires strategies such as incorporating self-healing mechanisms that repair damage, using tough and resilient network architectures, and minimizing stress concentrations through appropriate design. The interaction between mechanical fatigue and electrochemical cycling, which may involve coupled degradation mechanisms, represents an important consideration for energy storage applications [74,75,76].

### 3.3. Composite and Hybrid Polymer Hydrogels

The incorporation of functional additives into polymer hydrogel matrices to create composite and hybrid materials represents a powerful strategy for overcoming limitations of pure polymer hydrogels and achieving enhanced or multifunctional properties. Composite hydrogels can exhibit synergistic combinations of properties derived from both the polymer matrix and the incorporated additives, enabling optimization of characteristics including electrical conductivity, mechanical strength, electrochemical activity, and thermal management. The design of composite hydrogels requires careful consideration of multiple factors including additive selection, loading level, dispersion and distribution, interfacial interactions, and processing methods. Conductive nanomaterials including carbon nanotubes, graphene and its derivatives, carbon nanofibers, and metal nanoparticles represent the most widely investigated additives for enhancing the electrical conductivity of polymer hydrogels. These materials can create percolating networks within the hydrogel matrix that enable electronic conduction, transforming insulating polymer hydrogels into conductive composites suitable for electrode applications. The conductivity enhancement depends critically on the additive loading level, with a percolation threshold typically observed at loadings of a few percent by weight, above which conductivity increases dramatically. The aspect ratio and dispersion of conductive additives also strongly influence conductivity, with well-dispersed high-aspect-ratio materials such as carbon nanotubes and graphene sheets providing more efficient percolation networks [77,78,79].

**Figure 8:**
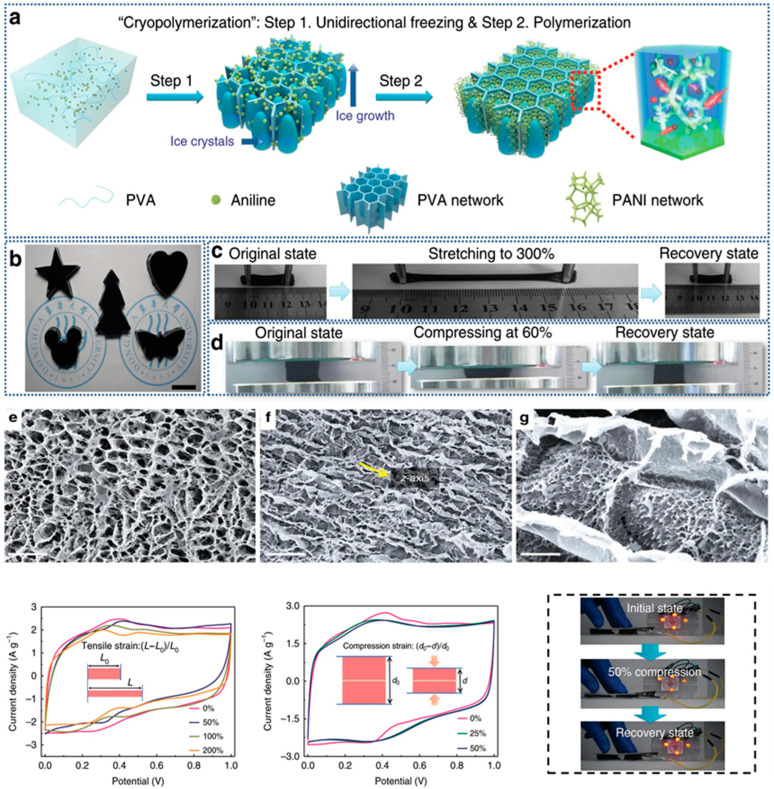
Cryopolymerization-Derived Anisotropic PVA/PANI Hydrogel Electrodes: (**a**) Fabrication Pathway, (**b**–**g**) Mechanical Deformation Behavior, and Supercapacitor Performance. SEM images of the APPH-2 from view of (**e**) perpendicular and (**f**,**g**) parallel to z-axis. Scale bars: (**e**,**f**) 20 μm, respectively; (**g**) 2 μm. Yellow arrow in f indicating the z-axis. [84].

Graphene and graphene oxide represent particularly attractive additives due to their exceptional electrical conductivity, high surface area, excellent mechanical properties, and versatility in chemical modification. Graphene oxide, with its oxygen-containing functional groups, can be readily dispersed in aqueous solutions and can participate in hydrogen bonding and other interactions with polymer chains, facilitating incorporation into hydrogel networks. Subsequent reduction of graphene oxide to graphene can be achieved through chemical, thermal, or electrochemical methods, enhancing electrical conductivity while potentially modifying the structure and properties of the composite. The loading level, degree of reduction, and distribution of graphene sheets critically influence the properties of graphene-polymer hydrogel composites (Figure 8). Metal oxide nanoparticles including manganese oxide, ruthenium oxide, and nickel oxide can provide both electrical conductivity and pseudocapacitive charge storage through reversible redox reactions, making them valuable additives for supercapacitor electrodes. The high theoretical capacitances of these materials, combined with the structural support and flexibility provided by the polymer matrix, can yield composite electrodes with excellent energy storage performance. However, challenges including aggregation of nanoparticles, limited electrical conductivity of some metal oxides, and potential dissolution or degradation during electrochemical cycling must be addressed through appropriate material selection and processing strategies [80,81,82,83].

MXenes, a relatively new class of two-dimensional materials consisting of transition metal carbides, nitrides, or carbonitrides, have emerged as promising additives for energy storage applications due to their combination of metallic conductivity, hydrophilic surfaces that facilitate aqueous processing, and electrochemical activity. MXene-polymer hydrogel composites can exhibit excellent electrical conductivity, high volumetric capacitance, and good mechanical flexibility. The layered structure of MXenes provides efficient pathways for ion transport, while the polymer matrix prevents restacking of MXene sheets that would reduce accessible surface area. Optimization of MXene loading, surface chemistry, and interaction with the polymer matrix is essential for achieving optimal composite properties.

Cellulose nanofibers and nanocrystals represent bio-derived additives that can enhance the mechanical properties of hydrogels while maintaining flexibility and biocompatibility. The high aspect ratio and strong mechanical properties of cellulose nanostructures enable effective reinforcement at relatively low loading levels. Additionally, the hydroxyl groups on cellulose surfaces can participate in hydrogen bonding with polymer chains, creating strong interfacial interactions that facilitate stress transfer. Cellulose-reinforced hydrogels can exhibit significantly improved mechanical strength and toughness while retaining high flexibility, making them attractive for wearable applications where mechanical robustness is critical.

The incorporation of conductive nanomaterials such as carbon nanotubes, graphene, carbon nanofibers, and metal nanoparticles into polymer hydrogels offers a powerful route to create multifunctional composite materials with enhanced electrical, mechanical, and thermal properties. However, a major challenge lies in preventing additive agglomeration, as poor dispersion can hinder network formation, reduce conductivity, and weaken mechanical integrity. Achieving uniform dispersion requires carefully chosen strategies, including surface functionalization of additives to improve compatibility with hydrophilic matrices, use of surfactants or dispersants to reduce surface tension, and physical methods such as ultrasonication or high-shear mixing to break apart clusters. In situ polymerization and interfacial coupling agents further promote stable filler distribution, while hybrid additive systems (e.g., CNT–graphene) prevent restacking and enhance percolation [80,81,82,83,84,85]. By combining these approaches, researchers can create homogenous conductive networks that reach the percolation threshold efficiently, ensuring the resulting hydrogels exhibit superior electrochemical and mechanical performance for applications in sensors, flexible electronics, and biomedical interfaces.

### 3.4. Sustainability and Biocompatibility

**Figure 9:**
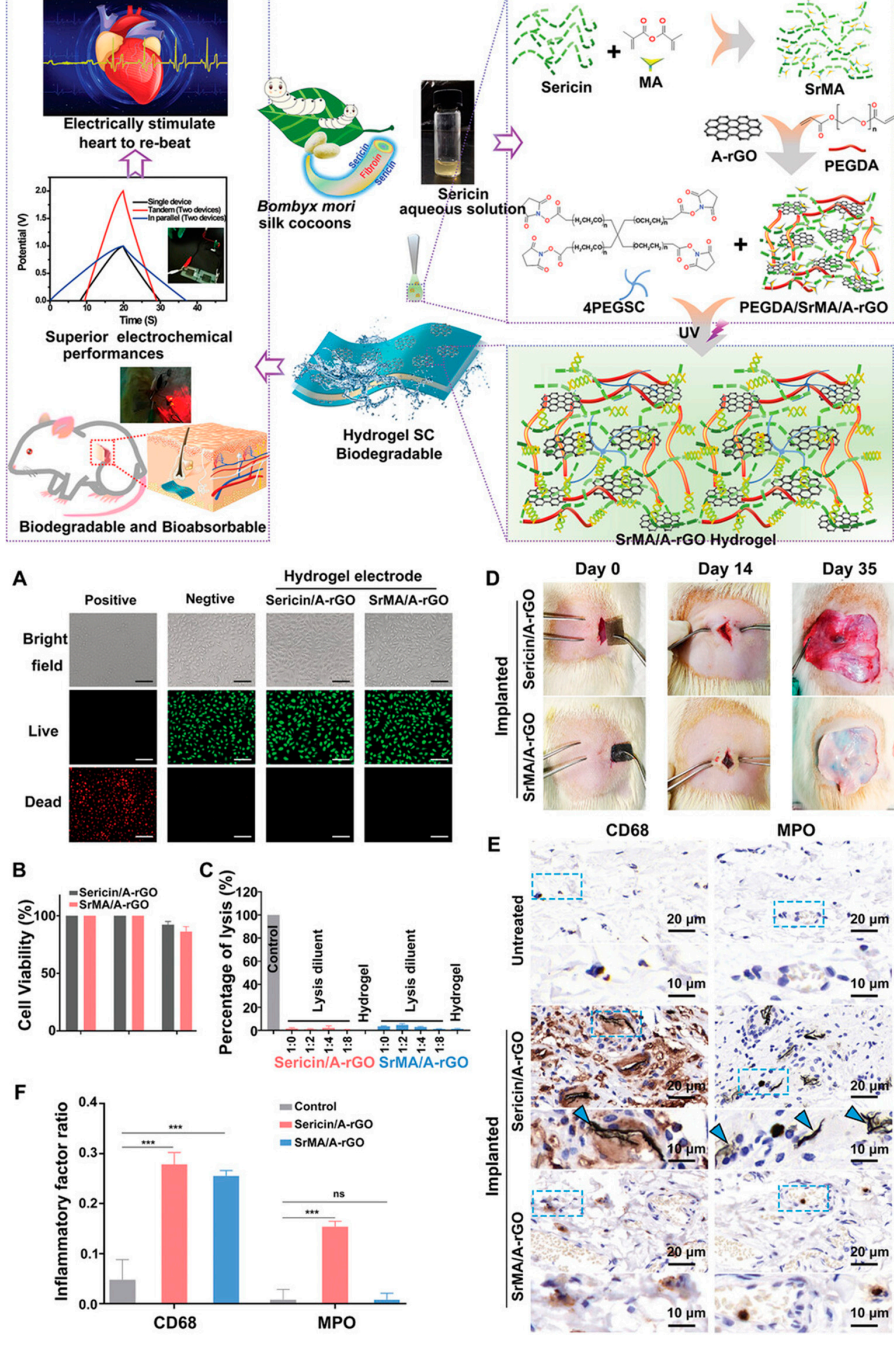
Schematic Fabrication and (**A**–**F**) Biological Performance of SrMA/A-rGO Hydrogel Electrodes in Energy Storage Devices [89].

The increasing awareness of environmental challenges and the growing interest in bio-integrated electronic devices have elevated sustainability and biocompatibility to critical considerations in the design of polymer hydrogels for energy storage applications. Sustainability encompasses multiple dimensions including the use of renewable or recycled raw materials, energy-efficient and low-impact synthesis processes, long device lifetimes that reduce replacement frequency, and end-of-life recyclability or biodegradability. Biocompatibility, defined as the ability of a material to perform its intended function without eliciting adverse biological responses, is essential for applications involving direct contact with biological tissues, including implantable devices and skin-worn electronics. Natural polymer hydrogels derived from biopolymers such as cellulose, chitosan, alginate, and gelatin offer inherent advantages in terms of sustainability and biocompatibility (Figure 9). These materials are obtained from renewable resources including plants, crustacean shells, seaweed, and animal tissues, and their production generally has lower environmental impact compared to synthetic polymers derived from petroleum. Many biopolymers are biodegradable through enzymatic or hydrolytic mechanisms, facilitating end-of-life disposal and reducing environmental persistence. Furthermore, natural polymers often exhibit excellent biocompatibility and low toxicity, having evolved in biological systems and consisting of chemical structures familiar to living organisms [85,86,87,88,89].

However, natural polymer hydrogels face challenges including batch-to-batch variability related to natural source variations, limited mechanical properties compared to many synthetic polymers, susceptibility to microbial degradation that can limit shelf life and operating lifetime, and generally low intrinsic electrical conductivity. Addressing these limitations while preserving sustainability and biocompatibility advantages requires strategies such as chemical modification to improve properties while maintaining biocompatibility, crosslinking to enhance mechanical properties and stability, and incorporation of conductive additives derived from sustainable sources. Synthetic polymers offer greater control over properties and performance but raise concerns regarding sustainability and biocompatibility. Many commodity synthetic polymers are derived from petroleum feedstocks and their production involves energy-intensive processes and potentially hazardous chemicals. Furthermore, most synthetic polymers are not biodegradable and can persist in the environment for extended periods. However, advances in green chemistry and sustainable polymer synthesis are enabling the development of bio-based synthetic polymers derived from renewable feedstocks such as plant oils, sugars, and amino acids. Additionally, the design of biodegradable synthetic polymers through incorporation of hydrolytically or enzymatically cleavable linkages represents a strategy for addressing end-of-life concerns, including environmental persistence, microplastic formation, recycling inefficiency, greenhouse gas emissions and toxicity issues [90,91,92].

The biocompatibility of synthetic polymers varies widely depending on chemical structure and the presence of potentially toxic components such as residual monomers, initiators, crosslinkers, or additives. Polymers intended for biomedical or skin-contact applications must undergo rigorous biocompatibility testing including cytotoxicity, sensitization, irritation, and systemic toxicity assessments. Some synthetic polymers including polyvinyl alcohol, polyethylene glycol, and certain polyacrylamides have demonstrated good biocompatibility and have received regulatory approval for biomedical applications. However, careful attention must be paid to purity and the absence of toxic residuals.

The sustainability and biocompatibility of composite hydrogels depend on both the polymer matrix and the incorporated additives. Carbon-based nanomaterials such as graphene and carbon nanotubes, while offering excellent electrical conductivity, raise concerns regarding potential toxicity related to their needle-like morphology and high aspect ratio. Metal and metal oxide nanoparticles may leach ions that could be toxic at elevated concentrations. Addressing these concerns requires strategies including use of biocompatible forms of nanomaterials, surface modifications to reduce toxicity, and encapsulation or stabilization within the polymer matrix to prevent leaching. The development of sustainable and biocompatible conductive additives, such as carbon materials derived from biomass or conductive polymers, represents an important research direction.

## 4. Energy Storage Applications of Polymer Hydrogels

The unique combination of properties offered by polymer hydrogels, including mechanical flexibility, high ionic conductivity, large surface area, and tunable electrochemical characteristics, positions these materials as enabling components for various energy storage technologies. Polymer hydrogels can function in multiple roles within energy storage devices, including as electrodes that directly participate in charge storage reactions, as electrolytes that facilitate ion transport between electrodes, as separators that prevent electronic contact between electrodes while permitting ionic conduction, and as binders or structural matrices that maintain electrode integrity. This section examines the application of polymer hydrogels in three major categories of energy storage: batteries, supercapacitors, and thermal energy storage systems.

### 4.1. Polymer Hydrogels in Batteries

Batteries represent electrochemical energy storage devices that convert chemical energy into electrical energy through reversible redox reactions occurring at two electrodes separated by an electrolyte. The development of flexible batteries for wearable electronics requires materials that can maintain electrochemical performance while undergoing mechanical deformations, a requirement that polymer hydrogels are uniquely positioned to address. Hydrogels can be incorporated into batteries as flexible electrolytes, as components of composite electrodes, or as multifunctional separators, each application leveraging different aspects of hydrogel properties. The use of polymer hydrogels as flexible solid or quasi-solid electrolytes addresses one of the critical challenges in flexible battery development: the prevention of electrolyte leakage while maintaining high ionic conductivity. Traditional liquid electrolytes, while offering excellent ionic conductivity, present safety concerns related to leakage, flammability, and potential corrosion. Polymer hydrogel electrolytes immobilize the liquid electrolyte within a three-dimensional polymer network, providing mechanical integrity and preventing leakage while retaining much of the ionic conductivity of liquid electrolytes. High water content of hydrogels enables efficient ion transport through the aqueous phase, while the polymer network provides structural support and mechanical flexibility [93,94,95].

Aqueous rechargeable batteries based on hydrogel electrolytes offer advantages including inherent safety due to the use of non-flammable aqueous electrolytes, environmental friendliness, and cost-effectiveness. Zinc-ion batteries, which utilize zinc metal anodes and various cathode materials with aqueous zinc salt electrolytes, have attracted significant attention due to the abundance and low cost of zinc, the high theoretical capacity of zinc metal, and the compatibility with aqueous electrolytes. Polymer hydrogel electrolytes for zinc-ion batteries must provide high zinc ion conductivity, maintain flexibility over a wide temperature range including subzero temperatures, and prevent or minimize zinc dendrite formation that can lead to short circuits and capacity fade.

**Figure 10:**
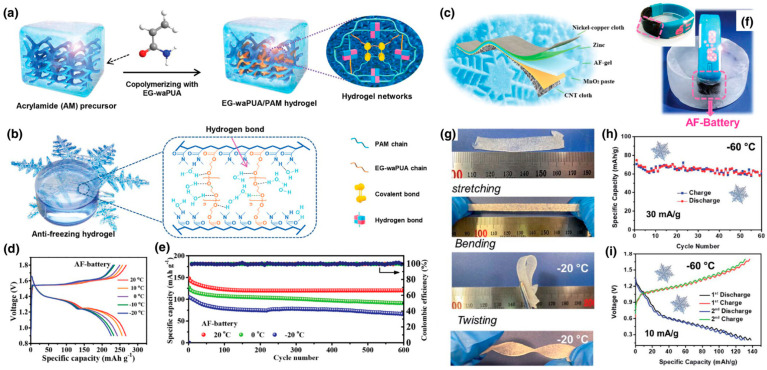
(**a**,**b**) Structural Design and (**c**–**i**) Low-Temperature Performance of Hydrogel-Based Flexible Batteries [96].

The design of hydrogel electrolytes with anti-freezing properties enables battery operation at low temperatures, expanding the potential application range. Strategies for achieving low-temperature performance include incorporation of anti-freezing agents such as glycerol or ethylene glycol that depress the freezing point of water, use of concentrated electrolyte solutions where strong ion-water interactions prevent ice formation, and creation of confined water environments within the hydrogel network where crystallization is kinetically hindered. These approaches can enable battery operation at temperatures as low as −40 °C while maintaining reasonable ionic conductivity and electrochemical performance (Figure 10). At elevated temperatures, hydrogel dehydration, polymer chain relaxation, and electrolyte decomposition become critical challenges affecting durability and safety. Most water-rich hydrogels begin to lose mass at >60 °C due to evaporation. Incorporation of hygroscopic salts (e.g., LiCl, ZnCl_2_) or ionic liquids enhances water retention up to 80–100 °C. When properly engineered with both anti-freezing and anti-dehydration mechanisms, hydrogel electrolytes demonstrate operational temperature between −40 °C to +100 °C; ionic conductivity between 10^−4^ and 10^−2^ S·cm^−1^; electrochemical stability window up to 2.0–3.0 V depending on electrolyte composition; mechanical resilience with a retention of >80% elastic modulus from −20 °C to 80 °C; and cyclic stability >90% capacity retention over 500–1000 charge–discharge cycles across −20 °C to 60 °C [96,97].

Lithium-ion batteries, the dominant technology for portable electronics, can also benefit from polymer hydrogel components, although the reactivity of lithium metal with water necessitates the use of non-aqueous or hybrid electrolyte systems. Hydrogels based on non-aqueous solvents or ionic liquids can provide flexibility and safety improvements while maintaining compatibility with lithium electrodes. Alternatively, hybrid electrolyte systems combining aqueous hydrogel separators with non-aqueous liquid electrolytes have been developed, leveraging the mechanical properties of hydrogels while avoiding direct contact between lithium and water. Polymer hydrogels can also function as components of flexible battery electrodes, either as binders that maintain the structural integrity of active material particles or as conductive matrices that facilitate electron transport. In composite electrodes, conductive polymer hydrogels or hydrogels containing conductive additives provide both mechanical support and electrical connectivity, while the porous structure accommodates volume changes in active materials during charge–discharge cycles. The flexibility of hydrogel-based electrodes enables the fabrication of batteries that can be bent, twisted, or stretched without performance degradation or structural failure [97,98,99].

### 4.2. Polymer Hydrogels in Supercapacitors and Electrochemical Capacitors

Supercapacitors, also known as electrochemical capacitors or ultracapacitors, store electrical energy through electrostatic charge accumulation at electrode-electrolyte interfaces and through fast surface or near-surface redox reactions. Compared to batteries, supercapacitors offer advantages including higher power density, faster charge–discharge rates, longer cycle life, and wider operating temperature ranges, although their energy density is typically lower. The development of flexible supercapacitors for wearable applications has been a major focus of research, with polymer hydrogels playing central roles as electrodes, electrolytes, and in all-hydrogel integrated devices where all components are based on hydrogel materials.

Polymer hydrogel electrodes for supercapacitors can provide charge storage through multiple mechanisms depending on the chemical nature of the hydrogel. Carbon-based hydrogels and polymer hydrogels containing conductive carbon additives store charge primarily through electric double-layer capacitance, where ions from the electrolyte accumulate at the electrode surface in response to applied voltage. The capacitance is proportional to the accessible surface area, making high-surface-area porous structures desirable. Conductive polymer hydrogels including polyaniline, polypyrrole, and poly(3,4-ethylenedioxythiophene) hydrogels store charge through pseudocapacitance, involving fast and reversible redox reactions at or near the electrode surface that provide higher specific capacitance than double-layer mechanisms. The design of high-performance hydrogel electrodes requires optimization of multiple properties including electrical conductivity to minimize resistive losses, high surface area to maximize charge storage capacity, appropriate pore structure to facilitate ion transport, mechanical flexibility to withstand deformations, and electrochemical stability over the operating voltage window. These requirements can be addressed through strategies such as incorporation of high-conductivity additives, creation of hierarchical porous structures combining macropores for ion transport with micropores for high surface area, and use of double crosslinked networks that combine mechanical robustness with flexibility [100,101,102,103].

Composite hydrogel electrodes combining conductive polymers with carbon nanomaterials can exhibit synergistic performance exceeding that of either component alone. The carbon nanomaterials provide high electrical conductivity and mechanical reinforcement, while the conductive polymer contributes pseudocapacitance and improves interfacial contact with the electrolyte. The porous structure of the hydrogel facilitates electrolyte penetration and ion transport, enabling efficient utilization of the active materials. Optimization of the composition, structure, and processing of these composite electrodes is essential for achieving high capacitance, good rate capability, and long cycle life.

Polymer hydrogel electrolytes for supercapacitors must provide high ionic conductivity to enable fast charge–discharge, a wide electrochemical stability window to allow operation at high voltages for increased energy density, mechanical flexibility and strength to maintain structural integrity during device deformation, and good interfacial contact with electrodes to minimize contact resistance. Polyvinyl alcohol-based hydrogel electrolytes with dissolved acids such as sulfuric acid or phosphoric acid represent a common class of materials that can achieve ionic conductivities exceeding 100 milliSiemens per centimeter. The addition of redox-active species to the electrolyte can further enhance energy density through additional faradaic charge storage. All-hydrogel supercapacitors, where electrodes, electrolyte, and separator are all based on hydrogel materials, represent an elegant approach to fully flexible energy storage devices. These integrated devices can be fabricated through sequential deposition or assembly of different hydrogel layers, or through single-step processes that create spatially differentiated hydrogel structures. All-hydrogel supercapacitors can exhibit remarkable mechanical properties including stretchability exceeding 1000 percent strain, self-healing capabilities that restore functionality after damage, and stable electrochemical performance under various deformation modes including bending, twisting, and stretching.

**Figure 11:**
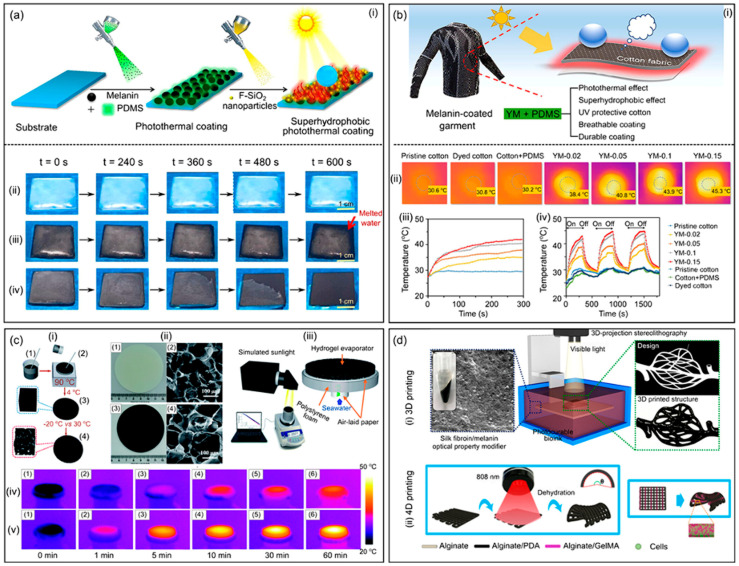
(**a**,**b**) Photothermal, (**c**) Deicing, and (**d**) Additive-Manufacturing Applications of Bio-Inspired Melanin-Based Materials [105].

The energy density of hydrogel-based supercapacitors, while typically lower than batteries, can be enhanced through strategies including increasing the operating voltage through use of wide-voltage-window electrolytes, maximizing the specific capacitance through optimization of electrode materials and structures, and employing asymmetric configurations where the positive and negative electrodes have different materials and capacitances optimized for their respective potential ranges. Recent advances have demonstrated hydrogel-based supercapacitors with energy densities approaching 100 Watt-hours per kilogram, narrowing the gap with batteries while maintaining the power and cycling advantages of supercapacitors (Figure 11) [101,102,103,104,105].

### 4.3. Polymer Hydrogels in Thermal Energy Storage

**Figure 12:**
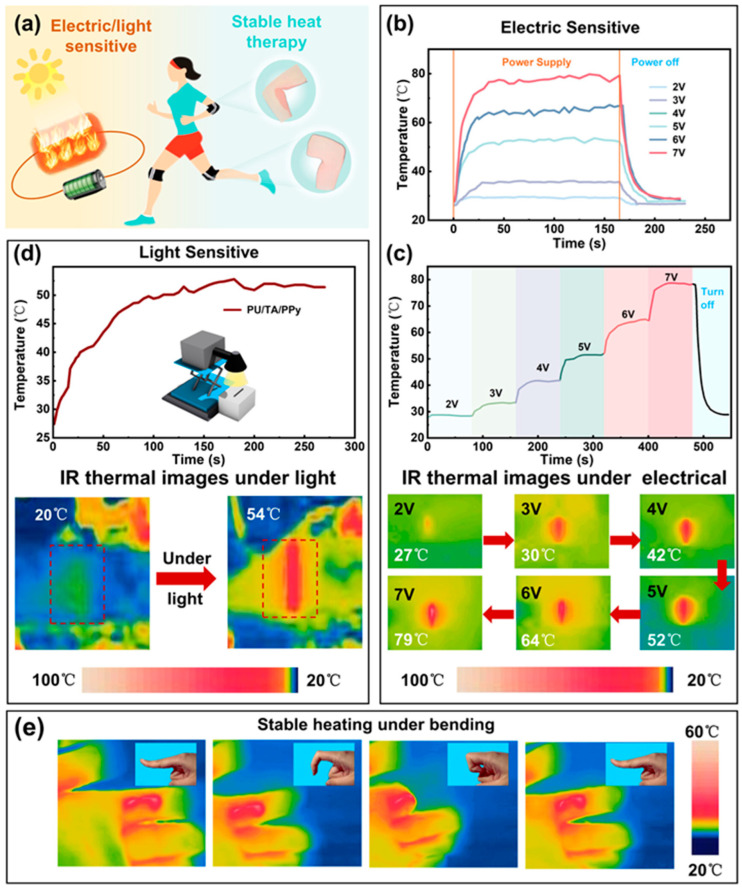
(a–e) Thermal Performance Characteristics of PU/TA/PPy Conductive Fibers [107].

Thermal energy storage systems capture and store thermal energy for later use, playing important roles in applications including building climate control, waste heat recovery, and thermal management of electronic devices. Phase change materials, which store and release large quantities of energy during melting and solidification at relatively constant temperatures, represent an effective approach to thermal energy storage. However, solid–liquid phase change materials face challenges including leakage in the liquid state, low thermal conductivity that limits charging and discharging rates, and lack of structural integrity. Polymer hydrogels can address these challenges by serving as shape-stabilizing matrices that encapsulate phase change materials, providing mechanical support while facilitating heat transfer. The design of hydrogel-based shape-stabilized phase change materials requires that the hydrogel matrix maintain the phase change material within its porous structure through capillary forces and interfacial interactions, preventing leakage even when the phase change material is molten. The hydrogel must possess sufficient porosity to accommodate a high loading of phase change material, as the energy storage capacity is directly proportional to the phase change material content. Additionally, the thermal conductivity of the composite system should be maximized to enable rapid heat transfer during charging and discharging cycles. The mechanical properties of the hydrogel must be sufficient to maintain shape stability during thermal cycling and potential mechanical loads. Various phase change materials have been incorporated into polymer hydrogel matrices, including paraffin waxes, fatty acids, polyethylene glycols, and salt hydrates. The selection of phase change material depends on the required phase transition temperature, which should match the application requirements, the latent heat of fusion that determines energy storage capacity, thermal conductivity, chemical stability, and compatibility with the hydrogel matrix (Figure 12). The loading of phase change material in the hydrogel can typically range from 50 to 90 percent by weight, with higher loadings providing greater energy storage capacity but potentially compromising mechanical properties and shape stability [105,106,107].

**Figure 13:**
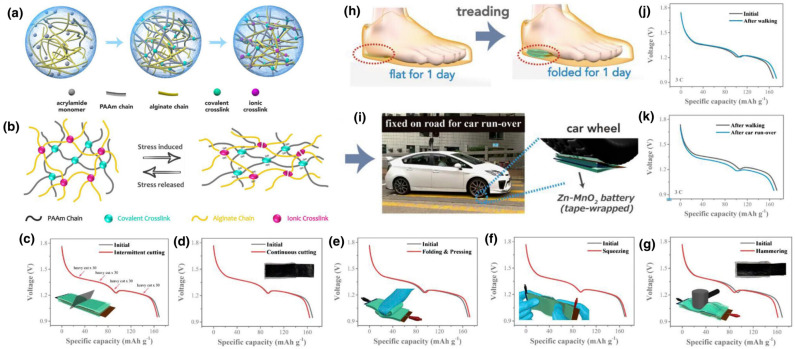
(**a**) Schematic; (**b**) Energy dissipation mechanism; and (**c**–**g**) Supercapacitor performance of hydrogel; (**h**–**k**) charge-discharge curve of hydrogel when placed under foot and car runover [109].

The thermal conductivity of polymer hydrogels is generally low, typically in the range of 0.1 to 0.5 Watts per meter per Kelvin, which can limit the rate of heat charging and discharging. Enhancement of thermal conductivity can be achieved through incorporation of thermally conductive additives such as graphene, carbon nanotubes, metal nanoparticles, or ceramic fillers. These additives create pathways for heat conduction through the hydrogel matrix, potentially increasing thermal conductivity by factors of two to ten depending on the additive type, loading, and dispersion. However, the addition of fillers must be balanced against potential reductions in phase change material loading and increases in material cost and complexity (Figure 13) [108,109]. A comparison table highlighting the performance of hydrogel-based battery systems is given in Table 2.

Polymer hydrogel-based thermal energy storage systems can also incorporate additional functionalities such as shape memory behavior, where the material can be deformed and fixed in a temporary shape, then recover its original shape upon heating through the phase transition temperature. This behavior can be exploited in applications including adaptive thermal management systems and smart textiles. The combination of thermal energy storage with other functions such as electrical conductivity for Joule heating or electromagnetic shielding represents another direction for multifunctional materials development.

## 5. Conclusions and Future Perspectives

Polymer hydrogels have emerged as versatile and promising materials for wearable energy storage applications, offering unique combinations of properties including mechanical flexibility, high ionic conductivity, tunable electrochemical characteristics, and potential for multifunctionality. This review has examined the fundamental aspects of polymer hydrogels including classification, synthesis methods, structure-property relationships, and applications in batteries, supercapacitors, and thermal energy storage systems. Significant progress has been achieved in recent years, with demonstrations of hydrogel-based devices exhibiting impressive performance metrics including high energy and power densities, excellent mechanical flexibility and stretchability, long cycle life, and self-healing capabilities.

Despite major progress in hydrogel-based wearable energy storage systems, future research must focus on meeting quantitative performance targets that enable practical deployment. To be commercially viable, next-generation hydrogel supercapacitors and batteries should aim for energy densities exceeding 10–20 mWh cm^−3^ and power densities above 50–100 mW cm^−3^, while maintaining mechanical stretchability of at least 200–500% without degradation. Long-term electrochemical stability should be demonstrated over >10,000 charge–discharge cycles with capacity retention above 90%, even under repeated bending or stretching. Furthermore, to ensure environmental and operational robustness, these devices must retain >85% performance after exposure to 80% relative humidity and temperature variations between −20 °C and 60 °C. In terms of scalability, synthesis routes should target material costs below $10 per m^2^ of active electrode area and processing times under 2 h per batch to enable industrial production. Achieving these metrics will require simplifying fabrication processes, developing low-cost conductive and biodegradable fillers, and improving polymer–nanomaterial interfaces to enhance both ionic conductivity and mechanical resilience under real-world conditions.

The environmental impact and sustainability of hydrogel-based energy storage systems deserve greater attention as these technologies move toward commercialization. While some hydrogel formulations based on natural polymers and benign additives offer excellent sustainability profiles, others employ synthetic polymers, toxic crosslinkers, or non-renewable additives that raise environmental concerns. The development of fully sustainable and biodegradable hydrogel energy storage systems, potentially based on bio-derived polymers and additives, represents an important direction for future research. Biodegradable networks formed through dynamic covalent bonds can provide mechanical durability while allowing reprocessing or environmental breakdown. Renewable fillers such as cellulose or lignin nanomaterials can enhance strength without relying on synthetic additives. Compostable device designs that pair biodegradable hydrogels with renewable carbon-based electrodes support environmentally friendly disposal.

The integration of polymer hydrogels into complete wearable systems requires consideration of factors beyond the materials themselves, including device architecture and packaging, electrical interconnections that maintain flexibility, compatibility with other device components such as power management electronics, and user interface considerations. The development of standardized testing protocols and performance metrics specific to flexible and wearable energy storage devices would facilitate comparison of results across different research groups and accelerate progress toward practical applications. Short-term goals should focus on improving nanomaterial dispersion within hydrogels, enhancing ionic and electronic conductivity (targeting > 10^−2^ S cm^−1^), and achieving high mechanical flexibility (≥300% stretchability) while maintaining toughness (>1 MJ m^−3^) and conductivity (>1 S m^−1^ under strain). Device durability should reach >5000 cycles with >90% capacitance retention under variable humidity and temperatures (−10 °C to 50 °C), and fabrication processes should be simplified to reduce material costs below $20/m^2^ and synthesis times under 2 h. The long-term goals should focus on multifunctional integration—combining energy storage with sensing and actuation—while ensuring environmental safety through biodegradable or bio-derived polymers that achieve >90% mass loss within 6 months under composting or enzymatic conditions. Future devices should maintain >10,000 stable charge–discharge cycles, achieve lifetimes exceeding 2 years under repeated deformation, and be produced through scalable roll-to-roll or 3D printing methods with at least 70% recyclability and 50% lower carbon emissions.

**Figure 14:**
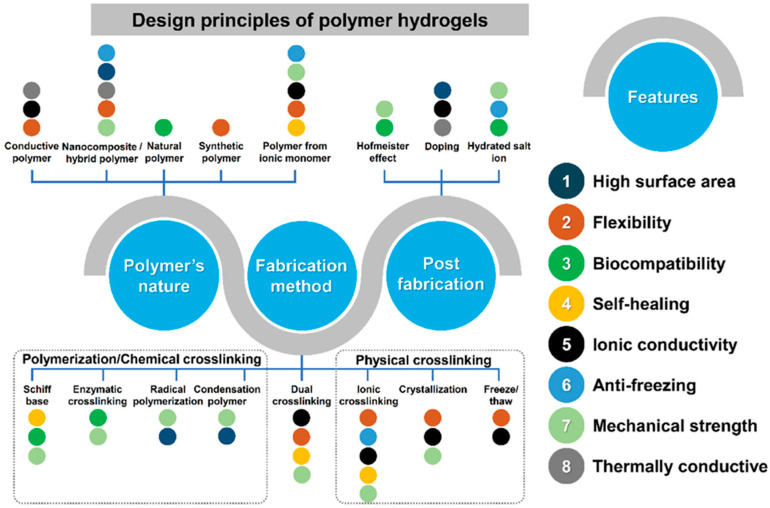
Key Design Strategies for Polymer Hydrogels to Meet Performance Requirements in Wearable Energy Storage Applications [13].

Looking forward, several emerging directions show particular promise for advancing hydrogel-based energy storage technologies. The development of multifunctional hydrogels that integrate energy storage with other capabilities such as sensing, actuation, self-healing, or adaptive properties could enable new classes of intelligent wearable devices. Hydrogels with strain- or pressure-dependent ionic pathways can enable real-time mechanical or motion sensing, while pH-, temperature-, or sweat-responsive conductivities offer built-in physiological monitoring. Electro-chemo-mechanical actuation, driven by ionic migration, could allow hydrogels to adapt their shape to the body during use. Materials incorporating phase-change domains may also provide thermoregulation while powering devices. Such multifunctional systems reduce the need for separate sensing layers, lowering device weight and complexity. Advanced manufacturing techniques including 3D printing, microfluidic synthesis, and roll-to-roll processing could enable scalable production of complex hydrogel structures with precisely controlled properties. 3D printing enables hydrogels with spatially graded mechanical or ionic properties, allowing more efficient and robust device architectures. Machine learning can accelerate formulation discovery by predicting optimal compositions for conductivity and stability. Microfluidic synthesis provides highly uniform nanogel components, while roll-to-roll printing offers continuous production of hydrogel films suitable for industrial-scale wearable energy devices (Figure 14). The convergence of advances in materials science, electrochemistry, mechanical engineering, and manufacturing technology positions polymer hydrogel-based energy storage systems for significant impact on wearable electronics and beyond. Continued research addressing the challenges outlined above, combined with increasing commercial interest and investment, suggests that hydrogel-based flexible energy storage devices will play important roles in the next generation of wearable technologies, potentially transforming how we interact with electronic devices and opening new possibilities for applications ranging from healthcare monitoring to augmented reality to environmental sensing.

## Figures and Tables

**Table 1:** Properties of Polymer Hydrogels obtained from Different Fabrication Methods.

Feature	Chemical Cross-Linking (Covalent)	Physical Cross-Linking (Non-Covalent)	Double Cross-Linking Network (DCN)	Ref.
Cross-linking Mechanism	Forms strong, irreversible covalent bonds. Requires a chemical cross-linking agent.	Relies on weaker, reversible non-covalent interactions (e.g., H-bonds, ionic forces).	A hybrid network combining a permanent chemical network and a dynamic physical network.	[32,36]
Key Advantages	High stability and structural integrity. Good electrochemical stability. Tunable network density.	Excellent self-healing capability due to reversible bonds. Simple, green fabrication. Good flexibility and stretchability.	Superior toughness and stretchability. Enhanced structural integrity and resilience under strain.	[46,69]
Key Disadvantages	Tends to be brittle at high cross-link density. Limited self-healing capability. May involve cytotoxic chemical reagents.	Generally lower mechanical strength. Lower stability under long-term or high-load operation. Potential for dissolution.	More complex and multi-step fabrication process. Balancing two networks can be challenging.	[36,61]
Mechanical Strength	Generally High (Stiff/Strong), but often brittle and with low resilience.	Generally Low (Soft/Weak), but can exhibit high elasticity and shear-thinning behavior.	Extremely High (Tough, Strong, and Resilient). Capable of absorbing significant energy.	[50,69]
Self-Healing Capability	Low/None. Fractures typically cause permanent damage.	High/Excellent. The dynamic bonds readily break and reform at the fractured interface.	High/Excellent. The physical network dissipates energy and facilitates micro-crack repair.	[46]
Conductivity Achievable	Good. Stable conductive path (e.g., PPy, PANI) but brittleness limits use under strain.	Moderate. Conductive components (ions, polymers) are easily integrated, but network stability is a challenge.	Excellent. The combination of high strength and dynamic bonds ensures the conductive path remains intact under high strain.	[54]
Typical Applications	Conventional tissue scaffolds, bio-adhesives, long-term sustained drug delivery systems.	Injectable drug delivery, soft hydrogel sensors, 3D bio-printing inks, cell culture matrices.	Flexible/Wearable Electronics (batteries, supercapacitors, sensors). Artificial cartilage, load-bearing tissue engineering.	[54]

**Table 2:** Performance Metrics of Different Hydrogel-Based Battery Systems.

Hydrogel System	Battery Type	Energy/Specific Capacity	Cycle Life/Capacity Retention	Flexibility	Ref.
Zwitterionic sulfobetaine–cellulose hydrogel	Zn–MnO_2_	275 mAh g^−1^ @ 1 C	90.42% retention @ 1200 cycles	Yes (920% strain)	[93]
PAMPS-k/MC hydrogel	Zn–air	764.7 mAh cm^−2^ @ 1 mA cm^−2^	Stable over 1000 cycles	Yes	[94]
PSBMA hydrogel	Zn metal	89 mAh g^−1^ @ 0.5 A g^−1^	83% retention @ 500 cycles; 99% CE @ 600 cycles	Yes (flexible + self-healing)	[95]
PAAm hydrogel electrolyte	Aqueous Zn battery	106 mAh g^−1^ @ 0.1 A g^−1^	100% retention @ 500 cycles	Yes	[97]
Cellulose/glycerol hydrogel	Zn–MnO_2_	230.1 mAh g^−1^ @ 1 A g^−1^	99.2% retention @ 2000 cycles	Yes (self-healable)	[98]
Chitosan/PVA hydrogel	Zn–EMD	310 mAh g^−1^ @ 0.1 A g^−1^	74% @ 50 cycles	Yes	[99]
M-HNT/PAAm hydrogel	Zn-ion	180.6 mAh g^−1^ @ 3.08 A g^−1^	92.7% @ 1000 cycles	Yes	[100]
AMP/Mn^2+^/PVA hydrogel	Zn–MnO_2_	95 mAh g^−1^ @ 2 A g^−1^	99.97% @ 600 cycles	Yes (self-healing)	[101]
RDC/N-PAA/KOH hydrogel	Zn–air	731.5 mAh cm^−2^ @ 2 mA cm^−2^	Excellent stability over 70 cycles	Yes	[102]
PAAm/ZnSO_4_/Gly/acetonitrile hydrogel	Zn-ion	185 mAh g^−1^ @ 5 A g^−1^	88% retention after 10,000 cycles	Yes	[103]
Antifreezing hydrogel Zn-ion system	Zn-ion	Capacity stable at −70 °C	High stability under bending; LED powered while bent	Highly flexible	[104]

## Data Availability

No new data were created or analyzed in this study.

## References

[1] Rohland P., Schröter E., Nolte O., Newkome G.R., Hager M.D., Schubert U.S. (2022). Redox-Active Polymers: The Magic Key towards Energy Storage—A Polymer Design Guideline. Prog. Polym. Sci..

[2] Zhang D., Lu J., Pei C., Ni S. (2022). Electrochemical Activation, Sintering, and Reconstruction in Energy-Storage Technologies: Origin, Development, and Prospects. Adv. Energy Mater..

[3] Xiao J., Han J., Zhang C., Ling G., Kang F., Yang Q.-H. (2022). Dimensionality, Function and Performance of Carbon Materials in Energy Storage Devices. Adv. Energy Mater..

[4] Shen Z., Zhang Z., Zhang N., Li J., Zhou P., Hu F., Rong Y., Lu B., Gu G. (2022). High-Stretchability, Ultralow-Hysteresis Conducting Polymer Hydrogel Strain Sensors for Soft Machines. Adv. Mater..

[5] Nazari N., Allahbakhsh A., Bahramian A.R. (2022). Analytical Effective Thermal Conductivity Model for Colloidal Porous Composites and Nanocomposites Based on Novolac/Graphene Oxide Aerogels. Int. J. Energy Res..

[6] Lage-Rivera S., Ares-Pernas A., Abad M.-J. (2022). Last Developments in Polymers for Wearable Energy Storage Devices. Int. J. Energy Res..

[7] Liu Y., Zhou H., Zhou W., Meng S., Qi C., Liu Z., Kong T. (2021). Biocompatible, High-Performance, Wet-Adhesive, Stretchable All-Hydrogel Supercapacitor Implant Based on PANI@rGO/Mxenes Electrode and Hydrogel Electrolyte. Adv. Energy Mater..

[8] Ganobjak M., Malfait W.J., Just J., Käppeli M., Mancebo F., Brunner S., Wernery J. (2023). Get the light & keep the warmth-A highly insulating, translucent aerogel glass brick for building envelopes. J. Build. Eng..

[9] Gonthier J., Rilling T., Scoppola E., Zemke F., Gurlo A., Fratzl P., Wagermaier W. (2023). In Operando μCT Imaging of Silylated Silica Aerogels during Ambient Pressure Drying and Spring-Back. Chem. Mater..

[10] Wernery J., Mancebo F., Malfait W.J., O’Connor M., Jelle B.P. (2021). The economics of thermal superinsulation in buildings. Energy Build..

[11] Bossert M., Grosman A., Trimaille I., Souris F., Doebele V., Benoit-Gonin A., Cagnon L., Spathis P., Wolf P.-E., Rolley E. (2021). Evaporation process in porous silicon: Cavitation vs pore blocking. Langmuir.

[12] Doebele V., Benoit-Gonin A., Souris F., Cagnon L., Spathis P., Wolf P.-E., Grosman A., Bossert M., Trimaille I., Rolley E. (2020). Direct observation of homogeneous cavitation in nanopores. Phys. Rev. Lett..

[13] Mahdavian F., Allahbakhsh A., Bahramian A.R., Rodrigue D., Tiwari M.K. (2023). Flexible Polymer Hydrogels for Wearable Energy Storage Applications. Adv. Mater. Technol..

[14] Zemke F., Gonthier J., Scoppola E., Simon U., Bekheet M.F., Wagermaier W., Gurlo A. (2023). Origin of the Springback Effect in Ambient-Pressure-Dried Silica Aerogels: The Effect of Surface Silylation. Gels.

[15] Peles-Strahl L., Honig H.C., Persky Y., Cullen D.A., Dahan A., Elbaz L. (2023). Modular Iron−Bipyridine-Based Conjugated Aerogels as Catalysts for Oxygen Reduction Reaction. ACS Catal..

[16] Wang L., Hu X., Huang R., Huang M., Liu X., Zhou Z., Guo P., Mao Z., Xu X., Wang X. (2024). Elastic, antiflaming phenolic aerogels for advanced thermal protection at extreme environments. Compos. Commun..

[17] Olad A., Pourkhiyabi M., Gharekhani H., Doustdar F. (2018). Semi-IPN superabsorbent nanocomposite based on sodium alginate and montmorillonite: Reaction parameters and swelling characteristics. Carbohydr. Polym..

[18] Gotad P.S., Kafle N., Miyoshi T., Jana S.C. (2022). Meso-and macroporous polymer gels for efficient adsorption of block copolymer surfactants. Langmuir.

[19] He L., Ling K., Wang M., Zhang X., Li J., Chen Y., Fan Y. (2024). Improving the phase change properties of paraffin wax by regulating the pore structure and surface properties of silica aerogel. J. Energy Storage.

[20] Li D., Cao Z.-W., Xie X.-Q., Chen X., He Y.-L. (2024). Experiments and numerical study on heat transfer of moist silica aerogel composites at high temperatures. Energy Storage Sav..

[21] Asadi N., Pazoki-Toroudi H., Del Bakhshayesh A.R., Akbarzadeh A., Davaran S., Annabi N. (2021). Multifunctional hydrogels for wound healing: Special focus on biomacromolecular based hydrogels. Int. J. Biol. Macromol..

[22] Dang W., Li Z., Wang B., Xu Z., Zhang X., Li F., Zhao K., Ma J., Tang Y. (2024). ZrO_2_/SiO_2_/C nanofiber aerogels with enhanced mechanical and thermal insulation properties fabricated by freeze casting. Ceram. Int..

[23] Ansari M.J., Rajendran R.R., Mohanto S., Agarwal U., Panda K., Dhotre K., Manne R., Deepak A., Zafar A., Yasir M. (2022). Poly(N-Isopropylacrylamide)-Based Hydrogels for Biomedical Applications: A Review of the State-of-the-Art. Gels.

[24] Sun Z., Zhao K., Yang H., Liang J., Chen Z., Feng J., Jiang Y., Li L., Hu Y., Feng J. (2024). Research Progress on Modification of Aerogels by Chemical Vapor Deposition. Langmuir.

[25] Valdez-Cano R., González-López J., Guerra-Cossío M. (2023). Effects on the Mechanical and Thermal Behaviors of an Alternative Mortar when Adding Modified Silica Aerogel with Aminopropyl Triethox ysilane and PEG-PPG-PEG Triblock Copolymer Additives. Silicon.

[26] Cai L., Shan G. (2015). Elastic silica aerogel using methyltrimethox ysilane precusor via ambient pressure drying. J. Porous Mater..

[27] Galy T., Mu D., Marszewski M., Pilon L. (2019). Computer generated mesoporous materials and associated structural character ization. Comput. Mater. Sci..

[28] Peng H., Zhou Y., Tong Y., Song Z., Feng S., Bu X., He M. (2022). Ultralight Hierarchically Structured RGO Composite Aerogels Embedded with MnO2/Ti3 C2 Tx for Efficient Microwave Absorption. Langmuir.

[29] Wang Y.-Y., Zhang F., Li N., Shi J.-F., Jia L.-C., Yan D.-X., Li Z.-M. (2023). Carbon-based aerogels and foams for electromagnetic interference shielding: A review. Carbon.

[30] Wei L., Wu Z., Tang S., Qin X., Xiong Y., Li J., Ruiz-Hitzky E., Wang X. (2023). Tracheid-inspired nanoarchitectured carbon-based aerogels with ultra-compressibility for wearable piezoresistive sensors. Carbon.

[31] Yoon S., Kim M.J., Kim C., Kim Y., Lee B., Lee C.Y., Lim B., Lee J.H. (2024). Chain-like One-Dimensional Assembly of Mesoporous Silica Nanoparticles: An Approach To Improve Hydrogel Adhesion. Langmuir.

[32] Xu Q., Gao S., Diao Z., Li J., Shao M., Song L., Wang X., Zhang M., Zhao J., Zhang F. (2025). Phenolic Modified SiO2 Aerogel as a Hybrid Thermal Insulation Systems. Langmuir.

[33] Tsai Y.-H., Chanda K., Chu Y.-T., Chiu C.-Y., Huang M.H. (2014). Direct formation of small Cu2O nanocubes, octahedra, and octapods for efficient synthesis of triazoles. Nanoscale.

[34] Omar S., Omar M., Attia N.F., El-Subruiti G.M., Eltaweil A. (2024). Rational engineering and fabrication of efficient nanophotocatalysts based on ZnO-SrO-CdS for pharmaceutical pollutants based waste water degradation. Surf. Interfaces.

[35] Song L., Liu F., Zhu C., Li A. (2019). Facile one-step fabrication of carboxymethyl cellulose based hydrogel for highly efficient removal of Cr (VI) under mild acidic condition. Chem. Eng. J..

[36] Wang Y., Lv T., Yin K., Feng N., Sun X., Zhou J., Li H. (2023). Carbon Dot-Based Hydrogels: Preparations, Properties, and Applications. Small.

[37] Zhang W., Wang R., Sun Z., Zhu X., Zhao Q., Zhang T., Cholewinski A., Yang F., Zhao B., Pinnaratip R. (2020). Catechol-Functionalized Hydrogels: Biomimetic Design, Adhesion Mechanism, and Biomedical Applications. Chem. Soc. Rev..

[38] Hua M., Wu D., Wu S., Ma Y., Alsaid Y., He X. (2021). 4D Printable Tough and Thermoresponsive Hydrogels. ACS Appl. Mater. Interfaces.

[39] Chatterjee S., Hui P.C. (2021). Review of Applications and Future Prospects of Stimuli-Responsive Hydrogel Based on Thermo-Responsive Biopolymers in Drug Delivery Systems. Polymers.

[40] Du Y., Xu J., Fang J., Zhang Y., Liu X., Zuo P., Zhuang Q. (2022). Ultralight, highly compressible, thermally stable MXene/aramid nanofiber anisotropic aerogels for electromagnetic interference shielding. J. Mater. Chem. A.

[41] Tafreshi O.A., Ghaffari-Mosanenzadeh S., Karamikamkar S., Saadatnia Z., Kiddell S., Park C.B., Naguib H.E. (2022). Novel, flexible, and transparent thin film polyimide aerogels with enhanced thermal insulation and high service temperature. J. Mater. Chem. C.

[42] Sun J., Tan H. (2013). Alginate-based biomaterials for regenerative medicine applications. Materials.

[43] Teo N., Jana S.C. (2018). Solvent effects on tuning pore structures in polyimide aerogels. Langmuir.

[44] Wu B., Song X., Zheng D., Tan Q., Yao Y., Liu F.-Q. (2023). Wood inspired ultrafast high-performance adsorbents for CO_2_ capture. ACS Appl. Mater. Interfaces.

[45] Jana S., Kumar Sen K., Gandhi A. (2016). Alginate based nanocarriers for drug delivery applications. Curr. Pharm. Des..

[46] Jo Y.H., Li S., Zuo C., Zhang Y., Gan H., Li S., Yu L., He D., Xie X., Xue Z. (2020). Self-Healing Solid Polymer Electrolyte Facilitated by a Dynamic Cross-Linked Polymer Matrix for Lithium-Ion Batteries. Macromolecules.

[47] Kaur G., Kumar H., Singla M. (2022). Diverse applications of ionic liquids: A comprehensive review. J. Mol. Liq..

[48] Zhao Y., Wang F., Liu J., Gan D., Lei B., Shao J., Wang W., Wang Q., Dong X. (2023). Underwater self-healing and recyclable ionogel sensor for physiological signal monitoring. ACS Appl. Mater. Interfaces.

[49] Zurick K.M., Bernards M. (2014). Recent Biomedical Advances with Polyampholyte Polymers. J. Appl. Polym. Sci..

[50] Wu Y., Wang H., Yuan L., Zhang Q., Liu Y., Shao C., Hou Q., Sun R. (2024). Lignin-Based Functional Hydrogels: An Eco-Friendly Bulk Material. ACS Sustain. Chem. Eng..

[51] Deng F., Wang C., Xiang C., Wang R.J.N.E. (2021). Bioinspired topological design of super hygroscopic complex for cost-effective atmospheric water harvesting. Nano Energy.

[52] Li J., Xing G., Qiao M., Liu Z., Sun H., Jiao R., Li L., Zhang J., Li A. (2023). Guar Gum-Based Macroporous Hygroscopic Polymer for Efficient Atmospheric Water Harvesting. Langmuir.

[53] Abdulhameed A.S., Mohammad A.-T., Jawad A.H. (2019). Application of response surface methodology for enhanced synthesis of chitosan tripolyphosphate/TiO_2_ nanocomposite and adsorption of reactive orange 16 dye. J. Cleaner Prod..

[54] Arno M.C., Inam M., Weems A.C., Li Z., Binch A.L.A., Platt C.I., Richardson S.M., Hoyland J.A., Dove A.P., O’Reilly R.K. (2020). Exploiting the Role of Nanoparticle Shape in Enhancing Hydrogel Adhesive and Mechanical Properties. Nat. Commun..

[55] Pardo A., Gómez-Florit M., Barbosa S., Taboada P., Domingues R.M.A., Gomes M.E. (2021). Magnetic Nanocomposite Hydrogels for Tissue Engineering: Design Concepts and Remote Actuation Strategies to Control Cell Fate. ACS Nano.

[56] Wang X., Yang D., Zhang M., Hu Q., Gao K., Zhou J., Yu Z.-Z. (2022). Super-hygroscopic calcium chloride/graphene oxide/poly (Nisopropylacrylamide) gels for spontaneous harvesting of atmospheric water and solar-driven water release. ACS Appl. Mater. Interfaces.

[57] Yuan J., Liu X., Akbulut O., Hu J., Suib S.L., Kong J., Stellacci F. (2008). Superwetting nanowire membranes for selective absorption. Nat. Nanotechnol..

[58] Li H., Chen J., Chang X., Xu Y., Zhao G., Zhu Y., Li Y. (2021). A Highly Stretchable Strain Sensor with Both an Ultrarlow Detection Limit and an Ultrawide Sensing Range. J. Mater. Chem. A.

[59] Du K., Lin R., Yin L., Ho J.S., Wang J., Lim C.T. (2022). Electronic Textiles for Energy, Sensing, and Communication. iScience.

[60] Liu Q., Yi C., Chen J., Xia M., Lu Y., Wang Y., Liu X., Li M., Liu K., Wang D. (2021). Flexible, breathable, and Highly Environmental Stable Ni/PPy/PET Conductive Fabrics for Efficient Electromagnetic Interference Shielding and Wearable Textile Antennas. Compos. Part B.

[61] Lloyd E.C., Dhakal S., Amini S., Alhasan R., Fratzl P., Tree D.R., Morozova S., Hickey R.J. (2025). Porous Hierarchically Ordered Hydrogels Demonstrating Structurally Dependent Mechanical Properties. Nat. Commun..

[62] Van Den Bulcke A.I., Bogdanov B., De Rooze N., Schacht E.H., Cornelissen M., Berghmans H. (2000). Structural and rheological properties of methacrylamide modified gelatin hydrogels. Biomacromolecules.

[63] Hazarika A., Deka B.K., Jeong C., Park Y.B., Park H.W. (2019). Biomechanical Energy-Harvesting Wearable Textile-Based Personal Thermal Management Device Containing Epitaxially Grown Aligned Ag-Tipped-NixCo1−xSe Nanowires/Reduced Graphene Oxide. Adv. Funct. Mater..

[64] Deng B., Fang L., Fang K., Han X., Liang Y. (2023). Scalable Preparation of MWCNTs/PAN Conductive Composite Fibers with Tai Chi Structure for Thermotherapy Textiles. Compos. Sci. Technol..

[65] Gong M., Yue L., Kong J., Lin X., Zhang L., Wang J., Wang D. (2021). Knittable and Sewable Spandex Yarn with Nacre-Mimetic Composite Coating for Wearable Health Monitoring and Thermo and Antibacterial Therapies. ACS Appl. Mater. Interfaces.

[66] Chen Z.H., Fang R., Li W., Guan J. (2019). Stretchable Transparent Conductors: From Micro/Macromechanics to Applications. Adv. Mater..

[67] Gao Y., Yu L., Li Y., Wei L., Yin J., Wang F., Wang L., Mao J. (2022). Maple Leaf Inspired Conductive Fiber with Hierarchical Wrinkles for Highly Stretchable and Integratable Electronics. ACS Appl. Mater. Interfaces.

[68] Zheng L., Zhu M., Wu B., Li Z., Sun S., Wu P. (2021). Conductance-Stable Liquid Metal Sheath-Core Microfibers for Stretchy Smart Fabrics and Self-Powered Sensing. Sci. Adv..

[69] Yin B., Zhang S., Ke K., Wang Z. (2019). Advanced Deformable All-in-One Hydrogel Supercapacitor Based on Conducting Polymer: Toward Integrated Mechanical and Capacitive Performance. J. Alloys Compd..

[70] Ho M.D., Liu Y., Dong D., Zhao Y., Cheng W. (2018). Fractal Gold Nanoframework for Highly Stretchable Transparent Strain-Insensitive Conductors. Nano Lett..

[71] Sun F., Tian M., Sun X., Xu T., Liu X., Zhu S., Zhang X., Qu L. (2019). Stretchable Conductive Fibers of Ultrahigh Tensile Strain and Stable Conductance Enabled by a Worm-Shaped Graphene Micro layer. Nano Lett..

[72] Zarei M., Samimi A., Khorram M., Abdi M.M., Golestaneh S.I. (2021). Fabrication and Characterization of Conductive Polypyrrole/Chitosan/Collagen Electrospun Nanofiber Scaffold for Tissue Engineering Application. Int. J. Biol. Macromol..

[73] Zhang Y., Zhang W., Ye G., Tan Q., Zhao Y., Qiu J., Qi S., Du X., Chen T., Liu N. (2020). Core-Sheath Stretchable Conductive Fibers for Safe Underwater Wearable Electronics. Adv. Mater. Technol..

[74] Zhai W., Xia Q., Zhou K., Yue X., Ren M., Zheng G., Dai K., Liu C., Shen C. (2019). Multifunctional Flexible Carbon Black/Polydimethylsiloxane Piezoresistive Sensor with Ultrahigh Linear Range, excellent Durability and Oil/Water Separation Capability. Chem. Eng. J..

[75] Li Y., Gao Y., Lan L., Zhang Q., Wei L., Shan M., Guo L., Wang F., Mao J., Zhang Z. (2022). Ultrastretchable and Wearable Conductive Multifilament Enabled by Buckled Polypyrrole Structure in Parallel. npj Flex. Electron..

[76] Wang B., Cheng H., Zhu J., Yuan Y., Wang C. (2020). A Flexible and Stretchable Polypyrrole/Knitted Cotton for Electrothermal Heater. Org. Electron..

[77] Sun J., Huang Y., Fu C., Wang Z., Huang Y., Zhu M., Zhi C., Hu H. (2016). High-Performance Stretchable Yarn Supercapacitor Based on PPy@CNTs@Urethane Elastic Fiber Core Spun Yarn. Nano Energy.

[78] Lipomi D.J., Lee J.A., Vosgueritchian M., Tee B.C.-K., Bolander J.A., Bao Z. (2012). Electronic Properties of Transparent Conductive Films of PEDOT: PSS on Stretchable Substrates. Chem. Mater..

[79] Oh J.Y., Kim S., Baik H.-K., Jeong U. (2016). Conducting Polymer Dough for Deformable Electronics. Adv. Mater..

[80] Kaczmarek-Szczepańska B., Wekwejt M., Pałubicka A., Michno A., Zasada L., Alsharabasy A.M. (2024). Cold Plasma Treatment of Tannic Acid as a Green Technology for the Fabrication of Advanced Cross-Linkers for Bioactive Collagen/Gelatin Hydrogels. Int. J. Biol. Macromol..

[81] Wang Z., Kang H., Zhao S., Zhang W., Zhang S., Li J. (2018). Polyphenol-Induced Cellulose Nanofibrils Anchored Graphene Oxide as Nanohybrids for Strong Yet Tough Soy Protein Nanocomposites. Carbohydr. Polym..

[82] Xing L., Wang Y., Cheng J., Chen G., Xing T. (2023). Robust and Flexible Smart Silk/PEDOT Conductive Fibers as Wearable Sensor for Personal Health Management and Information Transmission. Int. J. Biol. Macromol..

[83] Wang Z., Zhao S., Huang A., Zhang S., Li J. (2019). Mussel-Inspired Codepositing Interconnected Polypyrrole Nanohybrids onto Cellu lose Nanofiber Networks for Fabricating Flexible Conductive Biobased Composites. Carbohydr. Polym..

[84] Li J., Wang F. (2022). Application of Conductive Polymer Hydrogels in Flexible Electronics. J. Polym. Sci..

[85] Zhou L., Fan L., Yi X., Zhou Z., Liu C., Fu R., Dai C., Wang Z., Chen X., Yu P. (2018). Soft Conducting Polymer Hydrogels Cross-Linked and Doped by Tannic Acid for Spinal Cord Injury Repair. ACS Nano.

[86] Abdi M.M., Azli N.F.W.M., Lim H.N., Tahir P.M., Karimi G., Hoong Y.B., Khorram M. (2018). Polypyrrole/Tannin Biobased Nanocomposite with Enhanced Electrochemical and Physical Proper ties. RSC Adv..

[87] Zhang Q., Qiao Y., Zhu J., Li Y., Li C., Lin J., Li X., Han H., Mao J., Wang F. (2021). Electroactive and Antibacterial Surgical Sutures Based on Chitosan-Gelatin/Tannic Acid/Polypyrrole Composite Coating. Compos. Part B.

[88] Nautiyal A., Qiao M., Cook J.E., Zhang X., Huang T.S. (2018). High Performance Polypyrrole Coating for Corrosion Protection and Biocidal Applications. Appl. Surf. Sci..

[89] Lv Q., Li X., Tian X., Fu D., Liu H., Liu J., Song Y., Cai B., Wang J., Su Q. (2023). A Degradable and Biocompatible Supercapacitor Implant Based on Functional Sericin Hydrogel Electrode. Adv. Energy Mater..

[90] Wang W., Chen F., Fang L., Li Z., Xie Z. (2022). Reversibly Stretchable Organohydrogel-Based Soft Electronics with Robust and Redox-Active Interfaces Enabled by Polyphenol-Incorporated Double Networks. ACS Appl. Mater. Interfaces.

[91] Wang J., Xu S., Du H., Zhang Z., Lv J., Sun Y., Wang L. (2023). Mechanism Research of SDBS-Functionalized Polypyrrole to Improve Electrochemical Performance of Screen-Printed Graphene Electrode. Electrochim. Acta.

[92] Chen Y., Shen L., Wang C., Feng S., Zhang N., Zhang K., Yang B. (2022). Utilizing Tannic Acid and Polypyrrole to Induce Reconstruction to Optimize the Activity of MOF-Derived Electro catalyst for Water Oxidation in Seawater. Chem. Eng. J..

[93] Mo F., Chen Z., Liang G., Wang D., Zhao Y., Li H., Dong B., Zhi C. (2020). Zwitterionic Sulfobetaine Hydrogel Electrolyte Building Separated Positive/Negative Ion Migration Channels for Aqueous Zn-MnO_2_ Batteries with Superior Rate Capabilities. Adv. Energy Mater..

[94] Sun N., Lu F., Yu Y., Su L., Gao X., Zheng L. (2020). Alkaline Double-Network Hydrogels with High Conductivities, Superior Mechanical Performances, and Antifreezing Properties for Solid-State Zinc-Air Batteries. ACS Appl. Mater. Interfaces.

[95] Leng K., Li G., Guo J., Zhang X., Wang A., Liu X., Luo J. (2020). A Safe Polyzwitterionic Hydrogel Electrolyte for Long-Life Quasi-Solid State Zinc Metal Batteries. Adv. Funct. Mater..

[96] Ma R., Xu Z., Wang X. (2023). Polymer Hydrogel Electrolytes for Flexible and Multifunctional Zinc-Ion Batteries and Capacitors. Energy Environ. Mater..

[97] Zhu M., Wang X., Tang H., Wang J., Hao Q., Liu L., Li Y., Zhang K., Schmidt O.G. (2020). Antifreezing Hydrogel with High Zinc Reversibility for Flexible and Durable Aqueous Batteries by Cooperative Hydrated Cations. Adv. Funct. Mater..

[98] Chen M., Chen J., Zhou W., Han X., Yao Y., Wong C.P. (2021). Realizing an All-Round Hydrogel Electrolyte toward Environmentally Adaptive Dendrite-Free Aqueous Zn-MnO_2_ Batteries. Adv. Mater..

[99] Xu P., Wang C., Zhao B., Zhou Y., Cheng H. (2021). A high-strength and ultra-stable halloysite nanotubes-crosslinked polyacrylamide hydrogel electrolyte for flexible zinc-ion batteries. J. Power Sources.

[100] Shen P., Hu Y., Ji S., Luo H., Zhai C., Yang K. (2022). A self-healing nanocomposite hydrogel electrolyte for rechargeable aqueous Zn-MnO2 battery. Colloids Surf. A.

[101] Quan Y., Zhou W., Wu T., Chen M., Han X., Tian Q., Xu J., Chen J. (2022). Sorbitol-modified cellulose hydrogel electrolyte derived from wheat straws towards high-performance environmentally adaptive flexible zinc-ion batteries. Chem. Eng. J..

[102] Ou X., Liu Q., Pan J., Li L., Hu Y., Zhou Y., Yan F. (2022). CO2-sourced anti-freezing hydrogel electrolyte for sustainable Zn-ion batteries. Chem. Eng. J..

[103] Hu Y., Shi R., Ren Y., Peng W., Feng C., Zhao Y., Zheng S., Li W., Sun Z., Guo J. (2022). A “Two-in-One” Strategy for Flexible Aqueous Batteries Operated at −80 °C. Adv. Funct. Mater..

[104] Zamarayeva A.M., Jegraj A., Toor A., Pister V.I., Chang C., Chou A., Evans J.W., Arias A.C. (2020). Electrode Composite for Flexible Zinc–Manganese Dioxide Batteries through In Situ Polymerization of Polymer Hydrogel. Energy Technol..

[105] Xie W., Dhinojwala A., Gianneschi N.C., Shawkey M.D. (2024). Interactions of Melanin with Electromagnetic Radiation: From Fundamentals to Applications. Chem. Rev..

[106] Koller-Hodac A., Leonardo D., Walpen S., Felder D. Knee Orthopaedic Device How Robotic Technology Can Improve Outcome in Knee Rehabilitation. Proceedings of the 2011 IEEE International Conference on Rehabilitation Robotics.

[107] Li W., Li Y., Shan M., Lan L., Hu M., Li J., Xuan W., Wang F., Wang L., Mao J. (2024). Durable Flexible Conductive Fiber Based on Cross-Linking Network Tannic Acid/Polypyrrole for Wearable Thermotherapy Monitoring System. ACS Appl. Mater. Interfaces.

[108] Park H., Kim J.W., Hong S.Y., Lee G., Lee H., Song C., Keum K., Jeong Y.R., Jin S.W., Kim D.S. (2019). Dynamically Stretchable Supercapacitor for Powering an Integrated Biosensor in an All-in-One Textile System. ACS Nano.

[109] Liu Z., Wang D., Tang Z., Liang G., Yang Q., Li H., Ma L., Mo F., Zhi C. (2019). A Mechanically Durable and Device-Level Tough Zn-MnO2 Battery with High Flexibility. Energy Storage Mater..

